# Trem2 deficiency differentially affects phenotype and transcriptome of human APOE3 and APOE4 mice

**DOI:** 10.1186/s13024-020-00394-4

**Published:** 2020-07-23

**Authors:** Nicholas F. Fitz, Cody M. Wolfe, Brittany E. Playso, Richard J. Biedrzycki, Yi Lu, Kyong Nyon Nam, Iliya Lefterov, Radosveta Koldamova

**Affiliations:** Department of Environmental & Occupational Health, University of Pittsburgh, 130 De Soto Street, Pittsburgh, PA 15261 USA

**Keywords:** *Trem2*, APOE, Transcriptomics, Microglia, Neuroinflammation, Alzheimer’s disease, Amyloid plaques, Neurodegeneration, APP transgenic mice, RNA-sequencing

## Abstract

**Background:**

Alzheimer’s Disease (AD) is a neurodegenerative disorder influenced by aging and genetic risk factors. The inheritance of *APOE*ε4 and variants of Triggering Receptor Expressed on Myeloid cells 2 (*TREM2*) are major genetic risk factors for AD. Recent studies showed that APOE binds to TREM2, thus raising the possibility of an APOE-TREM2 interaction that can modulate AD pathology.

**Methods:**

The aim of this study was to investigate this interaction using complex AD model mice - a crossbreed of Trem2^ko^ and APP/PSEN1dE9 mice expressing human APOE3 or APOE4 isoforms (APP/E3 and APP/E4 respectively), and their WT littermates (E3 and E4), and evaluate cognition, steady-state amyloid load, plaque compaction, plaque growth rate, glial response, and brain transcriptome.

**Results:**

In both, APP/E3 and APP/E4 mice, *Trem2* deletion reduced plaque compaction but did not significantly affect steady-state plaque load. Importantly, the lack of TREM2 increased plaque growth that negatively correlated to the diminished microglia barrier, an effect most pronounced at earlier stages of amyloid deposition. We also found that *Trem2* deficiency significantly decreased plaque-associated APOE protein in APP/E4 but not in APP/E3 mice in agreement with RNA-seq data. Interestingly, we observed a significant decrease of *Apoe* mRNA expression in plaque-associated microglia of APP/E4/Trem2^ko^ vs APP/E4 mice. The absence of TREM2, worsened cognitive performance in APP transgenic mice but not their WT littermates.

Gene expression analysis identified *Trem2* signature - a cluster of highly connected immune response genes, commonly downregulated as a result of *Trem2* deletion in all genotypes including APP and WT littermates. Furthermore, we identified sets of genes that were affected in TREM2- and APOE isoform-dependent manner. Among them were *Clec7a* and *Csf1r* upregulated in APP/E4 vs APP/E3 mice, a result further validated by in situ hybridization analysis. In contrast, *Tyrobp* and several genes involved in the C1Q complement cascade had a higher expression level in APP/E3 versus their APP/E4 counterparts.

**Conclusions:**

Our data demonstrate that lack of *Trem2* differentially impacts the phenotype and brain transcriptome of APP mice expressing human APOE isoforms. The changes probably reflect the different effect of APOE isoforms on amyloid deposition.

## Background

The inheritance of ε4 allele of apolipoprotein E (APOE) is the major genetic risk factor for late-onset Alzheimer’s disease (AD) [1, 2]. APOE is an apolipoprotein which, in the central nervous system, is secreted by glia; it facilitates the transport of cholesterol and phospholipids between cells [3, 4]. GWAS have identified TREM2 missense variants that are related to AD risk, with the largest risk conferred by the loss of function R47H variant [5–8]. TREM2 is a receptor of the innate immune system, expressed in mononuclear phagocytes, including microglia in brain [9]. The proteolytic cleavage of TREM2 generates soluble TREM2, which can be detected in CSF and has been proposed as a biomarker and shown to be increased in AD [10, 11]. Recent data showed that APOE can bind to TREM2, thus raising the possibility of an APOE-TREM2 interaction affecting TREM2 signaling [12–14]. Interestingly, Jendersen et al. showed that while all three human isoforms of APOE bind TREM2, APOE4 exhibits diminished interaction when compared to APOE2 and APOE3 [15].

In AD, microglia are important for the phagocytosis of debris, clearance of Aβ, release of pro-inflammatory cytokines, and development of a plaque-associated barrier [16]. Recently, Keren-Shaul et al. identified a subset of microglia named “disease-associated microglia” (DAM) that accumulate in AD and other neurodegenerative diseases [17]. DAM are characterized by the upregulation of genes involved in lysosomal, phagocytic, and lipid metabolism pathways, including genes known as AD risk factors, such as *APOE* and *TREM2* [18]. Simultaneously with the upregulation of DAM, Keren-Shaul et al. detected a significant downregulation of the so-called “homeostatic microglial” genes [17]. Furthermore, genetic ablation of *Trem2* suppressed mouse *Apoe* expression and restored homeostatic microglial function in AD-model mice [19]. This implicates TREM2 in the maintenance of the microglial response to amyloid pathology, further connecting APOE, TREM2, microglia function, and amyloid pathology. Loss of functional TREM2 in mice has been shown to increase plaque seeding, reduce plaque-associated microglia barrier, reduced plaque compaction, reduce the level of APOE in APP mice, and increased dystrophic neurites surrounding plaques [20–24]. The effect of TREM2 on amyloid deposition in AD mice is controversial, however, with some studies showing the lack of TREM2 increasing [25, 26] and others decreasing the amyloid load [19, 22]. Interestingly though, increased soluble TREM2 has been shown to increase microglia survival, reduce amyloid plaque load, increase microglia clustering and phagocytic activity in AD model mice [27, 28].

While APOE and TREM2 are two major genetic risk factors for LOAD, surprisingly little is known about the interplay between these two, regarding amyloid deposition, microglial phenotype or transcriptomic profile. In this study, we hypothesized that *Trem2* deletion would have a differential effect on the phenotype and transcriptome of APP and WT mice expressing human APOE3 and APOE4.

## Methods

### Experimental model and subject details

#### Animals

This study adhered to the guidelines outlined in the Guide for the Care and Use of Laboratory Animals from the United States Department of Health and Human Services and was approved by the University of Pittsburgh Institutional Animal Care and Use Committee. APP/PS1dE9 (B6.Cg-Tg (APPswe, PSEN1dE9)85Dbo/J) and *Trem2*^em2ADiuj^/J mice were purchased from The Jackson Laboratory (USA) and human *APOE3 (B6.129P2-Apoe*^tm3(APOE*3)Mae^*N8) and APOE4 (B6.129P2-Apoe*^tm3(APOE*4)Mae^*N8)* targeted replacement mice from Taconic (USA) [29]. APP/PS1dE9 mice express mutant familial variants of human amyloid precursor protein (APP) with Swedish mutation, and human presenilin 1 carrying the exon-9-deleted variant (PSEN1dE9). All purchased mice were on a C57BL/6 genetic background and crossbred for at least 10 generations in our laboratory.

APP/PS1dE9 mice were bred to human APOE3^+/+^ or APOE4^+/+^ targeted replacement mice [3, 30] to generate APP/PS1dE9/APOE3^+/+^ (referred to as APP/E3, APP/PS1dE9/APOE4^+/+^ (APP/E4), APOE3^+/+^ (E3), APOE4^+/+^ (E4) mice expressing human *APOE* isoforms and wild-type *Trem2*. Trem2^−/−^ mice were bred to human APP/PS1dE9/APOE3^+/+^ or APP/PS1dE9/APOE4^+/+^ targeted replacement mice to generate APP/PS1dE9/APOE3^+/+^/Trem2^−/−^ (referred to as APP/E3/Trem2^ko^), APP/PS1dE9/APOE4^+/+^/Trem2^−/−^ (APP/E4/Trem2^ko^), APOE3^+/+^/Trem2^−/−^ (E3/Trem2^ko^); and APOE4^+/+^/Trem2^−/−^ (E4/Trem2^ko^) mice. All *APOE3* or *APOE4* mice were littermates and fed normal mouse chow diet ad libitum. Mice had water accessible at all times and were kept on a 12-h light-dark cycle. Male and female mice from each genotype were used for this study at an average age of 6.5 months.

### Method details

#### Behavioral testing

Novel object recognition (NOR) was performed as previously described [31] with minor modifications. The NOR task assesses recognition memory and is based on the spontaneous tendency of mice to explore a novel object over a familiar one. Mice were placed in individual containers before any testing then returned to their housing cages after the daily testing was completed. Each mouse was handled for 3 min. for three successive days before testing to reduce anxiety. The NOR task was performed over three consecutive days, each pertaining to a unique phase. On Day 1, habituation phase, each animal was allowed to freely explore an open arena (40 cm X 40 cm X 30 cm tall white plastic box) for two 5 min. trials with a 5 min. inter-trial interval. On Day 2, familiarization phase, each animal was returned to the same arena for two 5 min. trials separated by a 5 min. intertrial interval with two identical objects (tower of LEGO® bricks 8 cm X 3.2 cm, built using white, blue, yellow, red, and green bricks) located in opposite diagonal corners of the arena. After a 24-h retention period, the testing phase was initiated on Day 3. The animal was returned into the arena with two objects in the same positions as the previous day, but one object was replaced with a novel object (metal bolt and nut of similar size). Mice were allowed to explore for one 10 min. interval. The exploration of both objects was recorded and scored with ANY-maze software (Stoelting Co., USA). The exploration by the software was defined as the mouse sniffing, climbing on, or interacting while facing an object within 3 cm. Mice were consistently placed into the middle of the arena facing the posterior wall to prevent any object preference. The arena and objects were cleaned with 70% ethanol between animals to prevent any olfactory cues. Animals that failed to have a total exploration time of 10 s for the objects during the novel phase were excluded from the analysis. The total distance traveled by each mouse was recorded during the habituation phase to assess locomotor activity. The percent exploration was determined by dividing the time exploring the novel object by the total time exploring both objects. This calculated value provides an indicator of recognition memory, with less time spent exploring the novel object signifying memory deficits.

Contextual and Cued Fear Conditioning (CCFC) was performed as previously described [31]. CCFC provides a measure of memory in relation to receiving a mild foot shock to a particular environment (context) or stimulus (cue). CCFC testing was initiated 24 h following completion of NOR and was performed for three consecutive days. On Day 1, training phase, mice were placed in a conditioning chamber (Stoelting Co., USA) for 5.5 min. The first 2 min. was silent, allowing the mouse to acclimate to the chamber. This was followed by a 30 s tone (2800 Hz; y 85 dB, conditioned stimulus (CS)) ending in a 2 s foot shock (0.7 mA, unconditioned stimulus (US)) through the floor of the conditioning chamber. The process was repeated one more time (learning phase) and ended with 30 s of reacclimation. On Day 2, contextual phase, mice were placed in the same conditioning chamber for 5 min. with no tone or shock administered, to measure contextual fear conditioning. For the final day, the gray walls of the chamber were covered with black and white striped walls to introduce a novel environment for assessing cued fear conditioning. Mice were placed in the conditioning chamber for 5 min. After the first 2 min. of silence (novel phase), the tone was administered for 3 min. (cued phase). Testing was performed at the same time of the day to ensure 24-h between phases. The chamber was cleaned with 70% ethanol between each animal. Freezing time was defined as the absence of movement except for respiration and recorded using ANY-maze software. Animals that had below 30 s total freezing time during the contextual phase were excluded from the analysis. Freezing time was calculated as percent freezing of the total time in the chamber during each phase of testing. Since freezing behavior is a fear characteristic in rodents, memory deficits were defined as diminished freezing when reintroduced to the context or cue from the training phase.

#### Animal tissue processing

Two days post behavior, mice were anesthetized by intraperitoneal injection using Avertin (250 mg/kg of body weight). Blood was collected from the right ventricle through a cardiac puncture, followed by transcardial perfusion through the left ventricle with 20 mL of cold 0.1 M phosphate buffered saline (PBS), pH 7.4. The brain was removed and divided into hemispheres. One hemisphere was dissected into the cerebellum, subcortical, hippocampus and cortex regions and flash frozen on dry ice. A separate section of the anterior cortex was removed for whole-brain RNA-seq. The other hemisphere was drop fixed in 4% phosphate-buffered paraformaldehyde at 4 °C for 48 h and stored in 30% sucrose until sectioning [3]. Hemibrains were mounted in O.C.T. and cut in the coronal plane at 30 μm sections using a frozen cryotome (Thermo Scientific, USA). Six serial sections were collected with each section 450 μm apart, starting approximately 150 μm caudal to the first appearance of the dentate gyrus; covering an area in the brain from bregma − 1.25 mm and ending at bregma − 3.95 mm. Sections were stored in glycol-based cryoprotectant at − 20 °C until histological staining [3].

#### Chemicals

Methoxy-X34 (X34) 1,4-bis (3-carboxy-4-hydroxyphenylethenyl)-benzene, was provided by William E Klunk, MD, PhD, (University of Pittsburgh). For in vivo labeling of dense plaques, we used Methoxy-X04 (X04) 1,4-bis (4′-hydroxystyryl)-2-methoxybenzene, synthesized in W. Klunk’s lab (University of Pittsburgh). X04 readily crosses the blood-brain barrier [32] and remains bound to plaques for at least 90 days [33]. One mg/mL X04 stock solution was made by dissolving X04 in a vehicle consisting of 4% DMSO and 7.7% Cremophore EL in phosphate buffered saline (PBS).

#### Histological staining

For the OC-X34 dual stain, free-floating sections were washed followed by 30 min incubation in 50 mM sodium citrate buffer at 80 °C for antigen retrieval. Sections were then washed, blocked with 5% normal horse serum made in 0.1% triton-X 100 PBS for 1 h and finally incubated in anti-OC (1:1000, Millipore) primary antibody overnight at 4 °C [34]. The following morning, the sections were washed, incubated in horse anti-rabbit Dylight 594 (Di-1094 Vector Labs) for 2 h, and washed before mounting on positively charged glass slides. The slides were treated with 100 μM X34 followed by two 5 min destaining steps in 50% ethanol and coverslipped. Fluorescent images were taken using a Nikon Eclipse 90i microscope at 10X magnification. OC-X34 images were analyzed using Nikon NIS elements software and thresholding for the detection of plaques. The ratio between X34 positive (compact plaque) and OC positive areas (protofibular Aβ) was used to determine plaque compaction. Plaques with increased OC / X34 ratio are less compact than plaques with a ratio closer to one. We also assessed the area of OC not associated with X34 positive plaques (non-core bound OC) as a percentage of total detectable OC area (total OC).

A second series of brain tissue was used for 6E10 immunostaining as previously described [3] with some modifications. Antigen retrieval was performed on free-floating sections using 70% formic acid, followed by quenching of endogenous peroxidases with 0.3% hydrogen peroxide. The tissue was incubated in 3% normal goat serum (Vector, USA) then blocked for endogenous avidin and biotin. Sections were incubated in 6E10 biotinylated antibody (1:1000 Biolegend, USA) for 2 h and subsequently developed using the Vector ABC kit and DAB substrate kit (Vector, USA). Sections were mounted onto superfrost plus slides (Fisher Scientific, USA) and coverslipped. Bright-field images were taken using a Nikon Eclipse 90i microscope at 4X magnification.

Thioflavin S (ThioS) staining was performed on a third series of brain sections. Sections were mounted onto slides, washed in PBS, and stained with 0.02% ThioS (Sigma, USA) in PBS for 10 min. Next, sections were differentiated in 50% ethanol for 2 min. After a final wash in PBS, slides were coverslipped. Fluorescent images were taken using a Nikon Eclipse 90i microscope at 4X magnification.

To quantify plaque pathology, two separate regions of interest (ROI) were drawn around the cortex and hippocampus for each section and an image intensity threshold was established to detect the stained plaques compared to the background using NIS Elements software (Nikon Instruments Inc., USA). OC, X34, 6E10, and ThioS staining values were represented as the area of staining normalized to ROI area or percentage of area covered by 6E10 or ThioS stain.

A forth series of brain sections were immunostained with anti-IBA1 antibody (WAKO, USA) and anti-GFAP antibody (Agilent, USA). Free-floating sections were washed, then antigen retrieval performed in sodium citrate buffer at 80 °C for 60 min, blocked in normal donkey serum (Jackson Lab, USA) for 1 h, and finally incubated in IBA1 antibody (1:1000) overnight at 4 °C. Sections were washed and transferred into secondary donkey anti-rabbit Alexa 594 (Invitrogen, USA) for 1 h, before being washed and transferred to GFAP antibody (1:1000) overnight at 4 °C. Again, sections were washed and transferred into secondary donkey anti-rabbit Alexa 488 (Invitrogen, USA) for 1 h, before being washed and mounted onto slides. Slides were stained with X34 as documented for ThioS, followed by DAPI staining, and coverslipped. Fluorescent images of individual plaques were taken using a Nikon Eclipse 90i microscope at 20X magnification. Plaques were chosen with an average area of 300 μm^2^. The number of IBA1 positive microglia and GFAP positive astrocytes were counted in circular radiating regions of interest with a diameter of 10, 20, 40, and 60 μm from the edge of the X34 positive plaque.

A fifth series of brain sections were immunostained with anti-APOE antibody (Invitrogen, USA) and Thiazine Red (TR, Sigma Aldrich). Free-floating sections were washed, blocked in normal donkey serum (Jackson Lab, USA) for 1 h, and incubated in APOE antibody (1:100) overnight at 4 °C. Sections were transferred into secondary donkey anti-rabbit Alexa 488 (Invitrogen, USA) for 1 h, before being washed and mounted onto slides. Mounted slides were stained with 2 μM TR in PBS for 15 min. After a final wash, slides were dried and coverslipped. Fluorescent images of plaques were taken using a Nikon Eclipse 90i microscope at 10X magnification. Staining was defined by threshold analysis using NIS Elements software, and the area of APOE staining associated with TR positive plaque area was assessed.

A sixth series of brain sections were immunostained with anti-LAMP1 antibody (Abcam, USA) and X34. Free-floating sections were washed, blocked in normal goat serum (Jackson Lab, USA) for 1 h, and incubated in LAMP1 antibody (1:500) overnight at 4 °C. Sections were transferred into secondary goat anti-rat Cy5 (Vector, USA) for 2 h, before being washed and mounted onto slides. Mounted slides were stained with X34 as above. Fluorescent images of plaques were taken using a Nikon Eclipse 90i microscope at 10X magnification. Staining was defined by threshold analysis using NIS Elements software, and the area of LAMP1 staining associated with X34 positive plaques was assessed. For all plaque specific imaging, plaques were selected so they were at least 50 μm from the edge of the tissue, and at least 50 μm away from other plaques, with an even representation of all plaque sizes and composition across all groups to account for any bias introduced by differences in plaque stage, composition, or size.

#### In vivo plaque labeling

X04 was administered intraperitoneally (i.p.) at a concentration of 10 mg/kg to mice at either 3.5 or 5.5 months of age and sacrificed after 30 days for the collection of brain tissue for in vivo labeling of dense core amyloid plaques. Tissues used for the X04-TR-IBA1 triple stain allowed for the assessment of plaque growth dynamics and microglia plaque barrier. Free-floating sections were washed, incubated in 0.5 uM TR in PBS for 20 min followed by a final PBS wash. Sections were then incubated in 50 mM sodium citrate buffer for 30 min at 80 °C to perform antigen retrieval, washed, incubated in 5% normal goat serum made in 0.1% triton-X 100 PBS for 1 h, and finally incubated in anti-IBA1 (1:1000, Wako) primary overnight at 4 °C. The following morning the sections were washed, incubated in goat anti-rabbit Dylight 488 (Di-1488 Vector Labs) for 2 h, and washed before mounting on positively charged glass slides. X04-TR-IBA1 triple stained tissues were imaged on all three channels using an Olympus FV1000 confocal microscope at 60x, with 1.5 μm step size. For confocal imaging, plaques were selected if they were at least 50 μm from the edge of the tissue, and at least 50 μm away from other plaques, with an even representation of all plaque sizes and composition across all groups.

To assess the size of β-amyloid plaques at each age group (4.5 and 6.5 months), we analyzed plaque volume using Imaris on an independent set of APP/E3, APP/E3/Trem2^ko^, APP/E4 and APP/E4/Trem2^ko^ mice to extract quantitative data from the high-resolution three-dimensional confocal images. In the 48 h controls, plaques of all sizes and compositions were intentionally imaged in order to account variance when thresholding near detection limits. Greater than 94% of all the plaques imaged in the experimental groups fall within the minimun and maximum range of TR plaque volume analyzed in the 48 h control group. Additionally, when comparing the TR volume to X04 volume in the 48 h control plaques we saw a very high correlation (R^2^ = 0.9579) and no departure from the linear regression line in either the extremely small, or extremely large plaques (see supplemental figure 3). Briefly, images were loaded into the Imaris (v9.3.1) environment and voxels less than 500 intensity were removed from all channels to reduce background noise. Surfaces were then generated for the X04 and TR channels and the volume of each surface created was analyzed. Surfaces were created to assess IBA1 coverage and, using the “surface-surface contact area” XTension, the percent of IBA1 / TR surface contact was calculated. A 1 voxel shell is generated surrounding the TR labeled amyloid plaque and any time IBA1 signal is colocalized with the TR shell it is counted as surface contact. The sum of the colocalized voxels divided by the total surface area of TR generated the percent surface contact. The surface area contacted by microglia is subtracted from the total surface area giving the exposed surface area of each plaque (surface area not covered by microglia). The change in plaque volume was calculated by subtracting the volume of the plaque at the time of sacrifice (TR) from the volume of the plaque at the time of in vivo labeling (X04). In the 48-h control mice, we found no significant difference in the volume of X04 and TR labeling, an average growth volume (TR-X04) near 0 and an average fold change near 1 (TR/X04).

#### Tissue homogenization for ELISA

The frozen cortices were homogenized according to previously published work [35]. Individual cortices were weighed and transferred into a glass Dounce containing the appropriate amount of tissue homogenization buffer (1 M TRIS base, 0.5 M EDTA, and 0.2 M EGTA) and protease inhibitor (Sigma-Aldrich, USA). Cortices were homogenized in 1 mL of tissue homogenization buffer and protease inhibitor per each 100 mg of tissue. Once homogenized, the tissue was spun in a centrifuge at *16,000 rcf f*or 1 h. The supernatant was kept for future use for the determination of soluble Aβ. 70% formic acid was then added to the pellet and sonicated for two fifteen sec intervals before being spun again at *16,000 rcf* for 1 h. The resulting supernatant was kept and used for the determination of insoluble Aβ.

#### Aβ ELISA

Aβ ELISA was performed according to previously published work [35] with modifications. The wells of MaxiSorp plates (Nunc) were coated by adding 100 μl/well of 6E10 antibody (Biolegend, USA) diluted to 5 μg/ml in Coating buffer (0.1 M NaHCO_3_, 0.1 M Na_2_ CO_3_, pH 9.6) and incubated overnight at 4 °C while rocking. The next day, wells were washed with PBS and 200 μL/well of Block Ace Solution (1% Block Ace (Bio Rad, USA) in PBS, 0.05% NaN_3_) was added. Plates were incubated for 4 h at room temperature with rocking to block non-specific binding. Once the Block Ace Solution was removed 50 μl/well of Buffer EC (20 mM sodium phosphate, 2 mM EDTA, 400 mM NaCl, 0.2% BSA, 0.05% CHAPS, 0.4% Block Ace, 0.05% NaN_3_, pH 7.0) was added to the wells to prevent drying while adding samples. 100 μL of standards and samples were added to each well, and high-range samples were diluted with Buffer EC where necessary. For the insoluble Aβ fraction, samples were also neutralized with FA neutralization solution (1 M TRIS base, 0.5 M Na_2_HPO_4_, 0.05% NaN_3_) before dilution. For standards, ranging from 0 to 0.8pM, equal parts of 8 pM stocks of Aβ40 and Aβ42 were used. Once standards and samples were loaded, the plates were incubated overnight at 4 °C with rocking. Plates were washed and 100 μL detection antibody HJ5.1 (1:3300, a gift from John Cirrito) diluted in 0.05%-PBSTween was added to each well and incubated for 90 min. at room temperature with rocking. After washing, 100 μL HRP40 secondary (1:16000, Fisher Scientific, USA) diluted in 1% BSA-PBSTween, was added to each well and incubated for 90 min. at room temperature with rocking. Wells were washed and 100 μL of prepared TMB Substrate/H_2_O_2_ Solution (Thermo Sci., USA) was added. Absorbance was read at 650 nm on a plate reader (Molecular Devices, USA). All samples were run on the same day in duplicates. The concentration of total Aβ in pM was interpolated using linear regression on GraphPad Prism 7.0 and multiplied by the dilution factor for each sample.

Bradford Assay. Protein concentrations were determined according to previously published work [36]. The Bradford assay was used to determine the protein concentration of all samples. Bovine Serum Albumin (BSA, Fisher Scientific, USA) standards ranging from 0 to 100 μg/ml were used. A 40% Bio-Rad Protein Assay Dye Reagent (Bio-Rad, USA) was prepared with a 1:1 volume of diluted samples and absorbance was read at 595 nm. Total Protein (μg/ml) was interpolated using linear regression on GraphPad Prism 7.0 and multiplied by the dilution factor for each sample. To normalize the data, total protein concentration from the Bradford assay (μg/ml) was divided by the pM concentration of total Aβ for each sample from ELISA.

#### Fluorescence in situ hybridization (FISH)

In a separate cohort, mice were perfused, and tissue fixed and sectioned as documented above. RNAscope experiments were performed using the Multiplex Fluorescent Reagent kit v2 (Advanced Cell Diagnostics, USA) following the manufacturer’s recommendations with minor adjustments. Six freshly sectioned tissues per animal were mounted onto superfrost plus slides (Fisher Scientific, USA) within a 0.75″ × 0.75″ square, and baked at 60 °C for 60 min. Slides were incubated in X34 for 10 min. before being dehydrated using a series of ethanol dilution steps, then submerged in target retrieval reagent at 100 °C for 10 min. Protease digestion was performed at 40 °C for 30 min. using Protease III, and probe hybridization was carried out at 40 °C for 2 h. We used probe sets available from ACD for *Clec7a*, *Csf1r*, *Apoe*, and *Tmem119*. Following the amplification steps, the sections were counterstained with DAPI and coverslipped. Imaging was carried out using a Nikon Eclipse 90i microscope at 20X magnification with imaging of individual plaques and analyzed using NIS Elements software (Nikon Instruments Inc., USA). One circular ROI that extends 50 μm from the center of the X-34 positive plaque was drawn, and a threshold established for each probe to determine the area of puncta coverage. Four ROI’s of the same area were randomly selected from areas away from the plaque on the same image and averaged to determine the area of puncta coverage greater than 50 μm from plaque center. *Apoe* FISH analysis was performed on *Tmem119*-positive microglia within the same 50 μm ROI. The nuclei and surrounding area of cells with positive *Tmem119* signal were outlined and identified as microglia. The intensity of *Apoe* FISH signal was normalized to the number of *Tmem119* positive cells.

#### mRNA-seq data

RNA was isolated from the frontal cortex and purified using RNeasy mini kit (Qiagen, Germany). RNA quality was assessed using 2100 Bioanalyzer (Agilent Technologies, USA) and only samples with RIN > 8 were used for library construction. Library generation was performed by Novogene Co. Ltd. and sequenced using an Illumina HiSeq 2500 instrument. Following initial processing and quality control, the sequencing data was aligned to the mouse genome mm10 using Subread/featureCounts (v1.5.3; https://sourceforge.net/projects/subread/files/subread-1.5.3/) with an average read depth of 50 million successfully aligned reads. Statistical analysis was carried out using Rsubread (v1.34.2; https://bioconductor.org/packages/release/bioc/html/Rsubread.html), DEseq2 (1.24.0; https://bioconductor.org/packages/release/bioc/html/DESeq2.html), EdgeR (v3.26.5; https://bioconductor.org/packages/release/bioc/html/edgeR.html), and WGCNA (v1.68; https://cran.r-project.org/web/packages/WGCNA/index.html) all in the R environment (v3.6.0; https://www.r-project.org/). For network analysis using WGCNA, samples were clustered by gene expression profile which enabled the detection of outliers that were removed from the downstream analysis. Modules were generated automatically using a soft thresholding power, β = 10, a minimum module size of 44 genes and a minimum module merge cut height of 0.25. To account for bias introduced by sequencing batch, we implemented empirical Bayes-moderated linear regression which removes variation in the data due to unwanted covariates while preserving variation due to retained covariates. Networks were built using the top 5 genes identified as hub genes from any given module (gene significance > 0.2, and module membership > 0.8). Following hub gene selection, all other connections generated from the top 5 genes were visualized using Cytoscape (v3.7.1). Functional annotation clustering was performed using the Database for Annotation, Visualization and Integrated Discovery (DAVID v6.8, https://david.ncifcrf.gov). All GO terms are considered significant if *p* < 0.05 following multiplicity correction using the Benjamini-Hochberg method to control the FDR.

### Quantification and statistical analysis

Sample sizes (n) indicated in the figure legends 1, 5, correspond to the number of biological replicates analyzed. Sample sizes (n) indicated in the figure legend 2, 3 correspond to the number of plaques analyzed from 6 biological replicates. No outliers were removed from the analysis. All researchers were blinded to experimental groups during the analysis. All results are reported as means ± SEM. Data was analyzed by two-way ANOVA with APOE isoform and *Trem2* status as main factors followed by Sidak multiple comparison test. Histology and FISH was analyzed by one-way ANOVA followed by Tukey’s multiple comparison test. Unless otherwise indicated, all statistical analyses were performed in GraphPad Prism (v 8.2.0), or in R (v 3.6.0) and significance was determined as p < 0.05. Number of experiments and statistical information are stated in the corresponding figure legends. In figures, asterisks denote statistical significance marked by * *p* < 0.05; ** *p* < 0.01; *** *p* < 0.001.

### Data and code availability

The RNA-seq expression data has been deposited in the GEO database under the accession number: GSE144125.

## Results

### *Trem2* deficiency worsens cognitive performance, affects plaque compaction, and impacts microglia recruitment in APP/E3 and APP/E4 mice

**Fig. 1:**
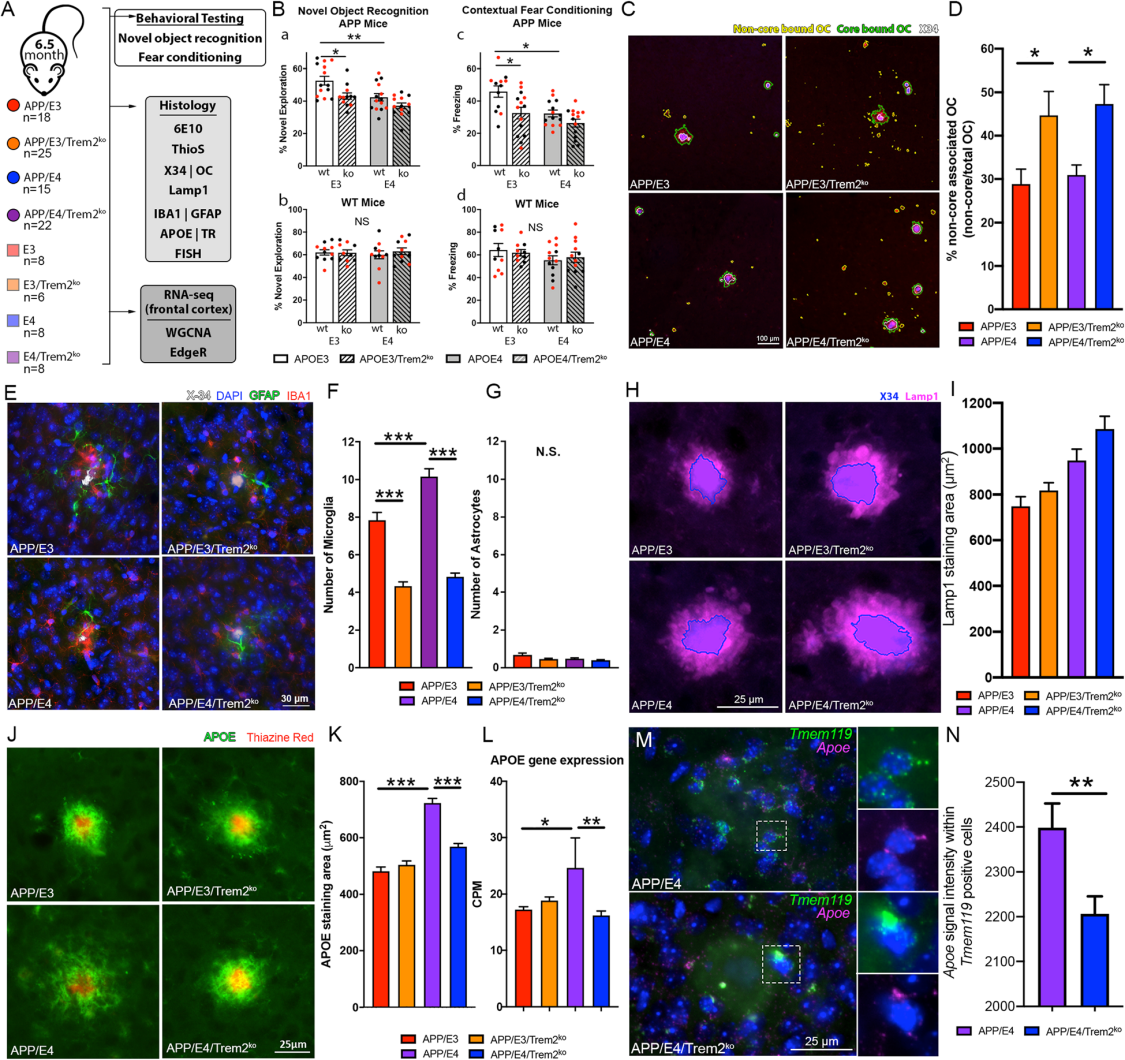
*Trem2* deficiency impacts cognition, plaque compaction, and microglia recruitment in APP/E3 and APP/E4 mice. **a** Schematic timeline showing groups and experimental procedures of 6.5-month-old mice used for behavioral, histological, and transcriptional analysis (Figs. 1, 4, 5, and 6). **b** Novel object recognition (NOR), and Contextual fear conditioning. Analysis by two-way ANOVA showed no interaction between APOE isoform and *Trem2* status and a significant main effect of APOE isoform (F (1, 49) = 13.28, *p* < 0.01) and *Trem2* status (F (1, 49) = 11.06, *p* < 0.01) for NOR (**a**, **b**), and Contextual fear conditioning (**c, d**) APOE isoform effect (F (1, 50) = 11.39, *p* < 0.01) and *Trem2* status (F (1,50) = 10.86, *p* < 0.01). ** *p* < 0.01; * *p* < 0.05, Sidak multiple comparisons test. *n* = 6–14 mice per group. For APP mice *n* = 6–7 mice/genotype/sex (12–14 mice/genotype). For non-APP mice, *n* = 4–7 mice/genotype/sex (8–14 mice/genotype). On the graphs, red symbols indicate female and black symbols indicate male mice. **c** Representative images of X34 and OC labeled amyloid deposits showing core-bound and non-core bound OC. **d** Bar plot depicting the ratio of non-core bound OC to total OC. *n* = 15–26 mice per group. **e** Representative images of glial cells (Iba1+ microglia and GFAP+ astrocytes) recruited to amyloid plaques. **f** Bar plots depicting the number of microglia nuclei within 60 μm of plaque border. **g** Bar plots depicting the number of astrocyte nuclei within 60 μm of plaque border. *n* = 80–120 plaques from 6 mice per group. **h** Representative images of X34 and LAMP1 label showing neuronal dystrophy surrounding amyloid deposits. X34 is shown as a blue region of interest defined by NIS elements thresholding. **i** Bar plot depicting the area of plaque-associated LAMP1 staining. Analysis by two-way ANOVA showed no interaction between APOE isoform and Trem2 status and a significant main effect of APOE isoform (F (1, 476) = 25.41, *p* < 0.0001) and Trem2 status (F (1, 476) = 4.99, *p* < 0.05) for LAMP1 area. Sidak multiple comparison test found no difference in plaque-associated LAMP1 staining area between APP/E3 vs APP/E3/Trem2^ko^ or APP/E4 vs APP/E4/Trem2^ko^. *n* = 120 plaques from 4 mice per group. **j** Representative images of plaque-associated APOE (green) and TR (red) staining to visualize compact amyloid plaques. **k** Bar plots showing the area of APOE staining that surrounds TR positive amyloid plaques. *n* = 874–2719 plaques from 4 to 6 mice per group. **l** Bar plots depicting *Apoe* gene expression as identified by RNA-seq, which closely follows the pattern of plaque-associated APOE protein levels. For histological analyses, one-way ANOVA was used followed by Tukey’s multiple comparison test. **m** Representative images of FISH analyses of gene expression near amyloid plaques (*Tmem119* – green, *Apoe* – Pink, Nuclei – Blue). **n** Bar plot depicting the *Apoe* gene expression within *Tmem119*-positive microglia cells. The intensity of *Apoe* FISH signal was normalized to the number of *Tmem119*-positive microglial cells. *n* = 279–313 microglia per group. Bars represent mean ± SEM, with all red bars = APP/E3, orange = APP/E3/Trem2^ko^, purple = APP/E4, and blue = APP/E4/Trem2^ko^. *** *p* < 0.001; ** *p* < 0.01; * *p* < 0.05; NS = No Signifincace

To determine the impact of *Trem2* deficiency on AD-like phenotype we used APP/PSEN1dE9 mice expressing human *APOE3* or *APOE4* genes (referred to as APP/E3, APP/E3/Trem2^ko^, APP/E4, and APP/E4/Trem2^ko^). For all behavioral and histological analysis we tested the mice at an average age of 6.5 months when amyloid pathology is readily detectable in mice expressing either APOE isoform and we previously have shown significant cognitive differences between APP/E3 and APP/E4 mice [30, 37]. The controls were age and gender-matched non-APP transgenic littermates expressing human *APOE3* or *APOE4* (referred to as E3, E3/Trem2^ko^, E4, and E4/Trem2^ko^) (Fig. 1a). To reveal differences in cognitive behavior, we used novel object recognition and contextual fear conditioning paradigms that demonstrated both factors - *Trem2* deficiency and APOE isoform, significantly affected cognition in APP mice, but not in their non-APP transgenic littermates (Fig. 1b). While *Trem2* deficiency was a significant factor in the behavioral performance, APP/E4 mice performed at the lower limits of both tasks and thus we were unable to observe a significant reduction in their Trem2^ko^ counterparts. The deterioration of memory was hippocampal-based as there was no significant difference during the amygdala-dependent cued phase (Suppl. Fig. 1A-B). There were no changes during the learning phase or novel phase of fear conditioning as well as no significant change in locomotor activity between genotypes (Suppl. Fig. 1C-H).

The examination of total amyloid (6E10, ThioS, X34, and OC staining) in cortex and hippocampus of the same 6.5 month mice assessed for cognitive changes revealed an effect of APOE isoform but no TREM2 effect (Suppl. Fig. 2A-G) - a result that was confirmed by ELISA (Suppl. Fig. 1I-J). To assess the proportion of compact to diffuse plaques we used X34 staining for compact amyloid and OC antibody that binds protofibrilar deposits [34]. As visible from Suppl. Fig. 2I-J, OC/X34 ratio was increased in Trem2^ko^ mice indicating reduced plaque compaction in both APOE isoforms caused by *Trem2* deletion. The assessment of OC outside dense amyloid cores (X34), determined that *Trem2* deletion significantly increased the percent of non-core bound OC (Fig. 1c-d, Suppl. Fig. 2H). Therefore, lack of *Trem2* did not affect total amyloid coverage but reduced plaque compaction and increased the presence of diffuse deposits which have not been sequestered into a dense core amyloid plaque.

To investigate if *Trem2* deficiency affects the number of glial cells recruited to amyloid plaques, brain sections were stained with IBA1 to label activated microglia and GFAP to label astrocytes (Fig. 1e-g). The lack of *Trem2* significantly reduced the number of microglia around the plaques in both APP/E3 and APP/E4 mice (Fig. 1f). Importantly, there was significantly more activated microglia in APP/E4 mice when compared to APP/E3, possibly reflecting the more advanced brain pathology of those mice. However, we did not find any difference between microglia numbers of APP/E3/Trem2^ko^ vs APP/E4/Trem2^ko^ mice suggesting that *Trem2* deletion blocks the conventional microglia response. Interestingly, there were very few astrocytes when compared to the number of microglia and their quantity was not significantly affected by *Trem2* deficiency (Fig. 1g).

To investigate if *Trem2* deficiency affects plaque-associated neuronal dystrophy we used immunostaining for LAMP1, a lysosomal protein enriched in dystrophic neurites [33, 38]. Analysis by two-way ANOVA demonstrated main effects of APOE and *Trem2* deficiency but no significant post hoc effect between Trem2^ko^ mice and their wild-type Trem2 counterparts (Fig. 1h-i).

Next, we determined the impact of *Trem2* deficiency on the level of APOE protein within the vicinity of amyloid plaques. We found that there was significantly more plaque-associated APOE in APP/E4 vs APP/E4/Trem2^ko^ mice, but *Trem2* deficiency did not impact APOE level in APP/E3 mice (Fig. 1j-k). This result correlated to the *Apoe* mRNA expression, as identified by RNA-seq. As shown on Fig. 1l, the lack of *Trem2* significantly affected *Apoe* expression level only in APP/E4 and not in APP/E3 mice or in their WT littermates (Suppl. Fig. 5B).

To determine the effect of *Trem2* deletion on *Apoe* expression in microglia, we used fluorescent in situ hybridization (FISH) to compare APP/E4 and APP/E4/Trem2^ko^ mice. Microglia were identified using the microglia-specific marker *Tmem119*. Our data demonstrated that *Apoe* mRNA expression is significantly higher in *Tmem119*-positive microglia surrounding amyloid plaques in APP/E4 vs APP/E4/Trem2^ko^ thus, validating the RNA-seq data (Fig. 1m-n). We conclude that the absence of *Trem2* similarly impairs microglia recruitment to plaques but has a differential effect on plaque-associated APOE protein and mRNA levels in APP/E3 and APP/E4 mice.

### *Trem2* deletion affects plaque growth depending on the stage of amyloid deposition

**Fig. 2:**
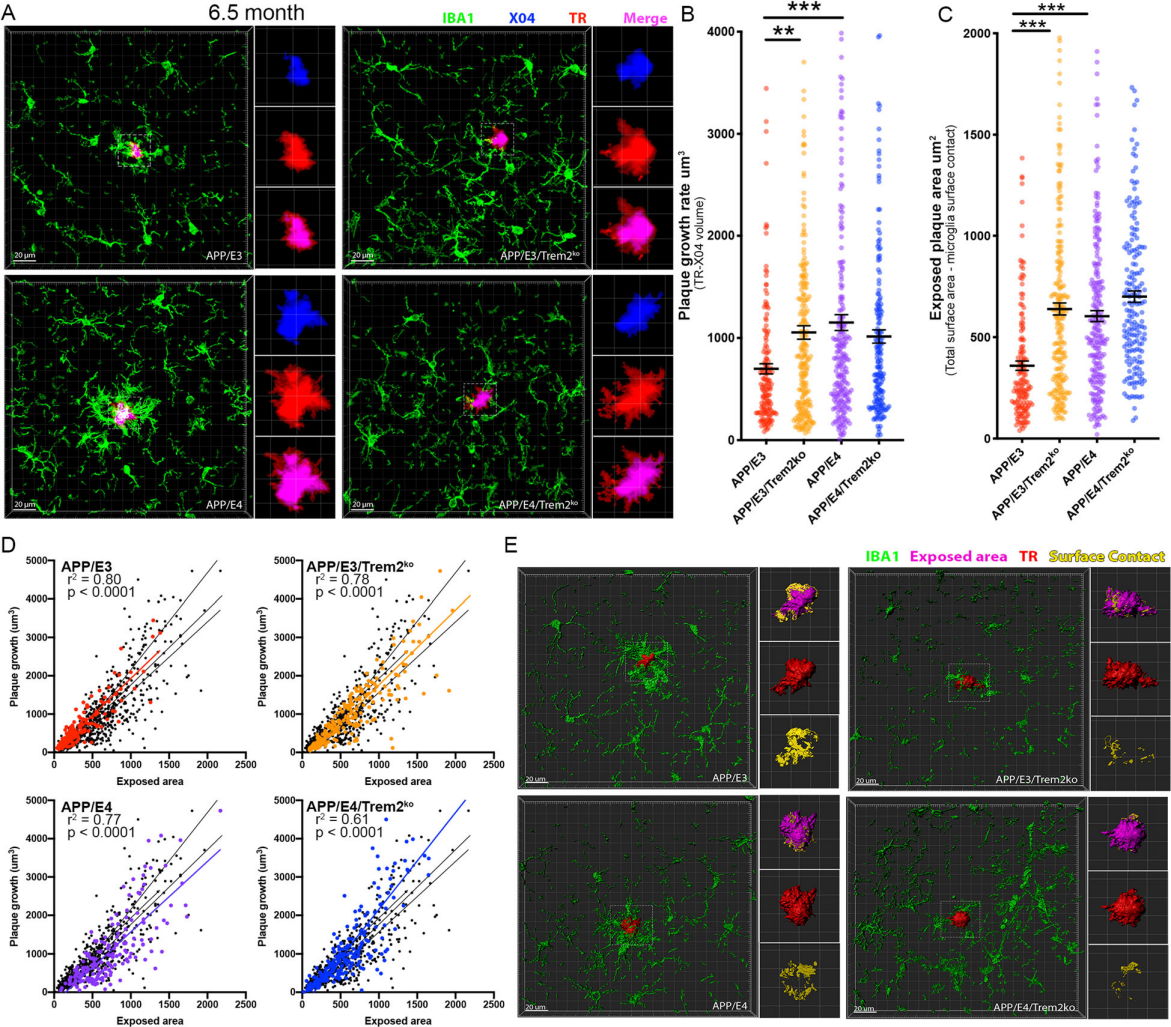
*Trem2* deletion affects plaque growth in correlation with microglia barrier at 6.5 months of age. **a** Representative confocal imaging of an amyloid plaque from the 6.5-month-old groups with IBA1 in green, X04 in blue, TR in red and the merge of X04-TR in pink. *n* = 6 mice/group (3 male and 3 female). APP/E3 *n* = 148, APP/E3/Trem2^ko^*n* = 219, APP/E4 *n* = 219, APP/E4/Trem2^ko^*n* = 180 individual plaques. **b** Quantification of the growth volume in 30 days for individual plaques. **c** The exposed area for each plaque is determined by the area in which microglia processes are not contacting the surface of TR (purple color in panel **e**). **d** Correlation between plaque growth and exposed surface area for each plaque and genotype. **e** Imaris generated 3D volumetric representations of an amyloid plaque from the 6.5-month-old group with IBA1 in green, exposed area in purple, TR in red, and surface contact in yellow. Analysis by one-way ANOVA followed by Tukey’s multiple comparison test. Bars represent mean ± SEM. *** *p* < 0.001; ** *p* < 0.01

**Fig. 3:**
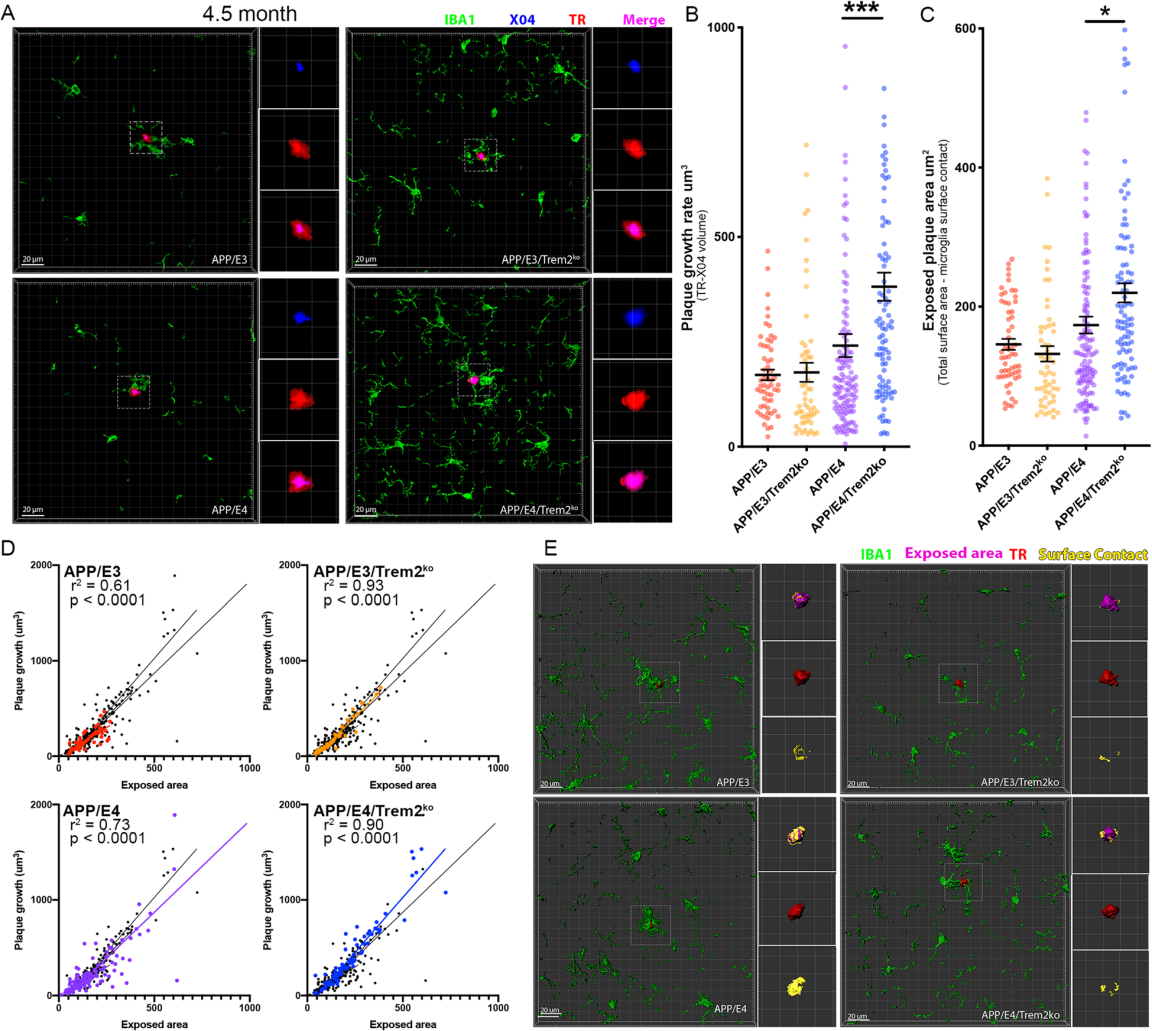
*Trem2* deletion affects microglia barrier and plaque growth at 4.5 months of age. **a** Representative confocal imaging of an amyloid plaque from the 4.5-month-old groups with IBA1 in green, X04 in blue, TR in red and the merge of X04/TR in pink. *n* = 6 mice per group (3 male and 3 female). APP/E3 *n* = 57, APP/E3/Trem2^ko^*n* = 54, APP/E4 *n* = 130, APP/E4/Trem2^ko^*n* = 94 individual plaques. **b** Quantification of the growth volume in 30 days for individual plaques. **c** The exposed area for each plaque is determined by the area in which microglia processes are not contacting the surface of TR (purple color in panel **e**). **d** Correlation between plaque growth and exposed surface area for each plaque and genotype. **e** Imaris generated 3D volumetric representations of an amyloid plaque from the 4.5-month-old group with IBA1 in green, exposed area in purple, TR in red, and surface contact in yellow. Analysis by one-way ANOVA followed by Tukey’s multiple comparison test. Bars represent mean ± SEM. * *p* < 0.05; *** *p* < 0.001

We showed that *Trem2* deficiency did not affect steady-state amyloid load (Suppl. Fig. 2). Here we evaluated whether the lack of *Trem2* affects the growth rate of individual amyloid plaques and if this correlates to the surrounding microglia barrier. To reveal this, we employed an in vivo labeling technique using the amyloid binding dye X04 followed by postmortem staining with TR [33]. Intraperitoneally injected X04 readily crosses the blood-brain barrier [32] and remains bound to plaques for at least 90 days post injection [33]. We injected the mice with X04 at 5.5 months of age and they were sacrificed 30 days later, followed by TR staining of sectioned tissues (Suppl. Fig. 3). Plaque growth was assessed using high-resolution confocal images in Imaris to generate 3D volumetric renderings of amyloid plaques by subtracting the volume of the plaque at the time of injection (X04 staining) from the volume of the plaque at the time of sacrifice (TR staining). For each individual plaque, IBA1 staining for activated microglia was used to determine the plaque surface area that is not covered by microglia i.e. “exposed” (see Suppl. Fig. 3H for details). As shown on Fig. 2a-b, *Trem2* deficiency significantly increased the growth of amyloid plaques in APP/E3 but not in APP/E4 mice. The examination of exposed plaque surface area follows the same pattern as plaque growth rate and is significantly affected only in APP/E3 mice (Fig. 2c&e). Subsequently, in all genotypes, we identified a very strong correlation between plaque growth rate and the exposed surface area suggesting that with the decrease of microglia barrier plaques grow faster (Fig. 2d). Our data also imply that *Trem2* deficiency may have a higher impact on plaque growth rate at earlier stages of amyloid deposition. Considering that amyloid deposition advances faster in APP/E4 mice and that there is a significant difference between the steady-state load of APP/E4 vs APP/E3 mice (Suppl. Fig. 2), it is possible that TREM2 affects APP/E4 plaque growth rate at an earlier age. To test this, we performed the same experiment in younger mice injected with X04 at 3.5 and sacrificed at 4.5 months (Fig. 3). Interestingly, we found that in the younger group, *Trem2* deficiency significantly increased amyloid plaque growth only in APP/E4 mice in agreement with significantly reduced microglia barrier around the plaques (Fig. 3). At this age, we observed that in contrast to APP/E4, age-matched APP/E3 had very little compact amyloid with almost no detectable X04 deposits in APP/E3/Trem2^ko^ mice that complicated the assessment of amyloid plaque growth in this genotype. Our data indicate that the absence of *Trem2* affects plaque growth depending on the stage of amyloid deposition and at different ages for APP/E3 and APP/E4 mice.

### Lack of *Trem2* significantly affects brain transcriptome in APOE3 or APOE4 mice

**Fig. 4:**
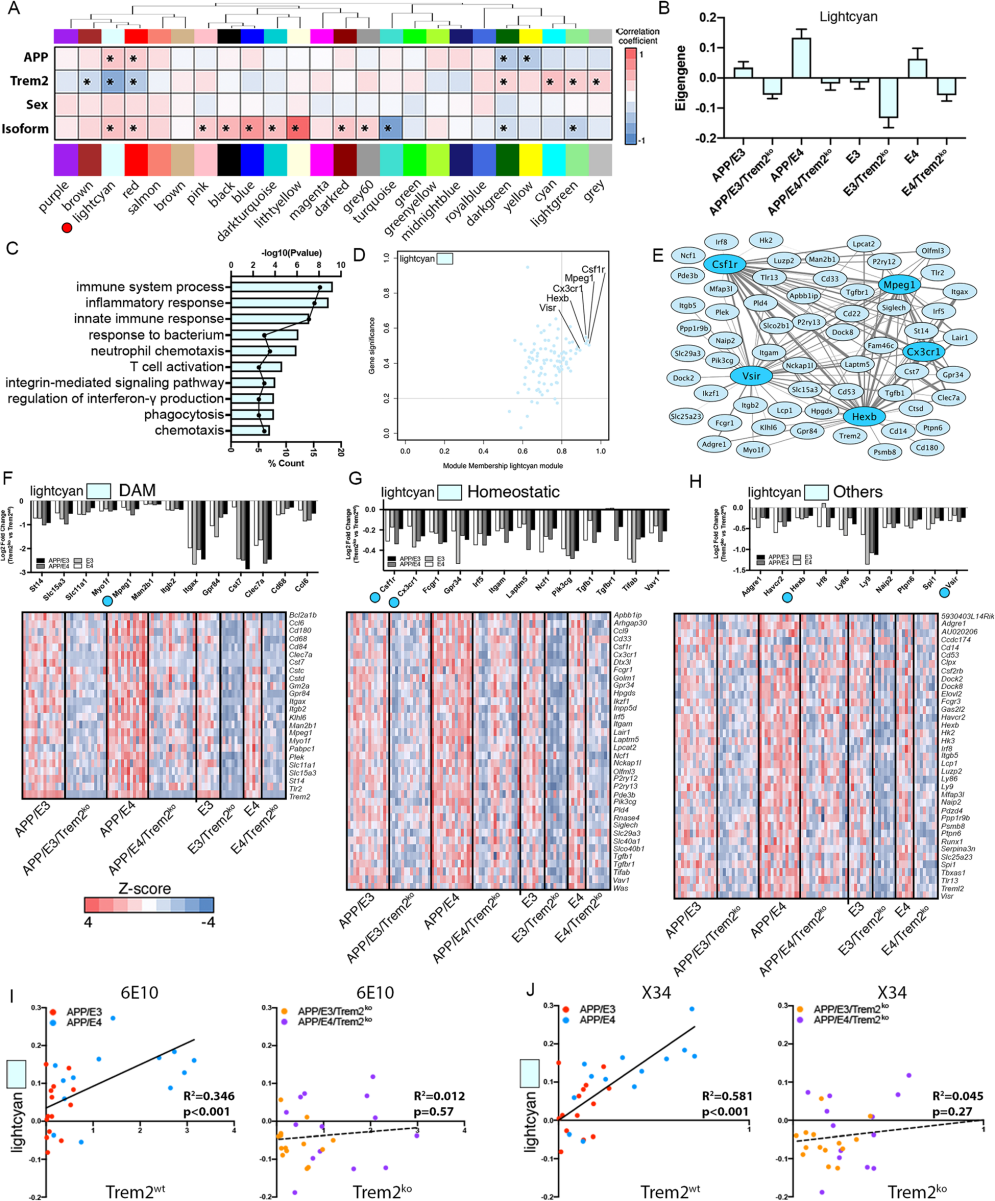
Lack of *Trem2* significantly affects brain transcriptome of mice expressing human APOE3 or APOE4. Gene expression profiling was performed by RNA-seq on tissue from the frontal cortex of the same 6–7 months old mice shown on Fig. 1a. **a** WGCNA was used to identify correlations between gene expression and four traits: APOE isoform, sex, *Trem2* genotype, and human APP transgene. The relationship table shows the correlation between the module eigengene (row) traits (column). Red denotes a positive correlation and blue a negative correlation, with * denoting a significant correlation (* *p* < 0.05). Trem2 signature module is marked with a red circle. **b** Bar plots show the aggregated module eigengene for each genotype in the modules of interest. **c** GO term bar plots indicate the -log10P value for each term. The associated point in the center of each bar represents the percent of submitted genes found in each GO term. **d** Scatterplot depicts the MM vs GS plot for genes in the lightcyan module relating to the *Trem2* genotype, with hub genes defined as MM > 0.8 and GS > 0.2. **e** The network generated from all connections within the module from the top 5 hub genes (indicated by darker blue color). Characterization of the lightcyan module with fold change bar plots and heatmaps for disease associated microglial genes (DAM) (**f**), homeostatic (**g**) microglia genes and genes outside these two categories (others) (**h**). Heatmaps depict the Z-score for genes downregulated in Trem2^ko^ mice from the lightcyan module. n: E3/Trem2^ko^ = 8; E4/Trem2^ko^ = 8; E3 = 8; E4 = 6; APP/E3/Trem2^ko^ = 14; APP/E4/Trem2^ko^ = 16; APP/E3 = 14; APP/E4 = 14; (equal number males and females**). i-j** Integration of co-expressed gene network of interest to amyloid deposition. The expression profile of lightcyan was used to identify correlations between gene expression patterns in each of the four APP groups to the percent coverage of 6E10 (**i**), and percent coverage of X34 (**j**). Correlation between histological data and RNA-seq data is done with Pearson r correlation

The effect of *Trem2* deficiency on brain transcriptome was examined by mRNA-seq on cortical tissue from all 8 genotypes shown on Fig. 1a at 6.5 months of age. First, we used weighted gene co-expression network analysis (WGCNA) to correlate gene expression to four traits - *Trem2* deficiency, APOE isoform, APP transgene/amyloid deposition and sex (Fig. 4a). The top three most significant modules correlated either to *Trem2* deficiency (lightcyan) or APOE isoform (turquoise and lightyellow). The turquoise module negatively correlates with APOE4 isoform and is associated with biological processes such as transport, translation, and mRNA processing and oxidation-reduction (Suppl. Fig. 4, Suppl. Table 1). The lightyellow module positively correlates with APOE4 isoform and biological processes associated with it are related to intermediate filament organization, immune system process, and innate immune response (Suppl. Fig. 4, Suppl. Table 1). In addition to lightyellow, another APOE isoform-specific module (darkturquoise see Suppl. Table 1) was positively associated with APOE4 isoform and represented GO terms such as acute-phase response, cholesterol efflux, and response to cytokines. Interestingly, this module contained *Apoe* and several members of *Serpina* family that were previously reported by us [39] and others [40] to be increased in an APOE4 dependent manner.

**Fig. 5:**
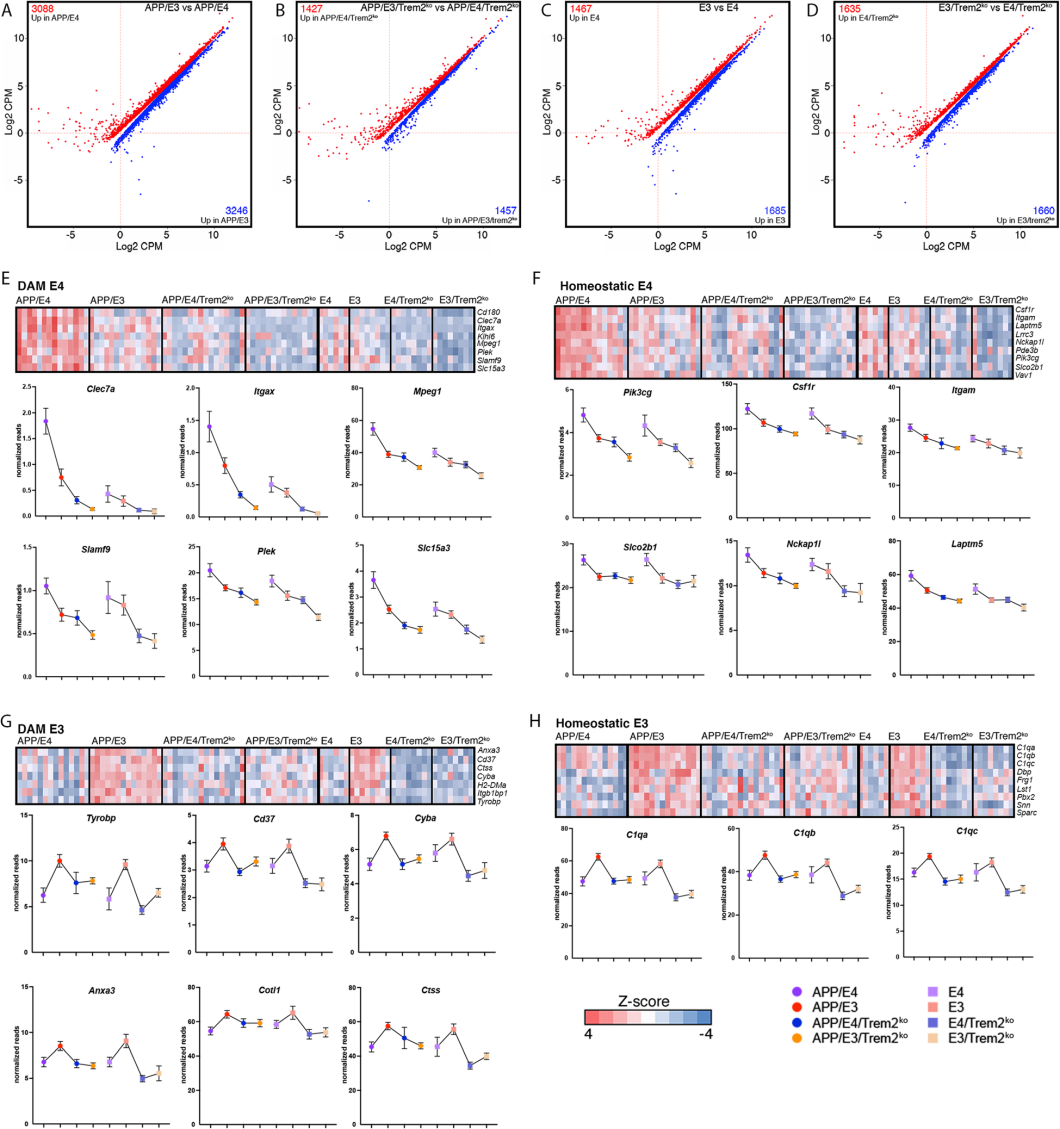
APOE isoform-specific effect on gene expression. RNA-seq data shown in Fig. 4 were analyzed using edgeR to identify differentially expressed genes between APOE isoforms. Scatterplots depict differentially expressed genes between APP/E3 vs APP/E4 (**a**), APP/E3/Trem2^ko^ vs APP/E4/Trem2^ko^ (**b**) E3 vs E4 (**c**), and E3/Trem2^ko^ vs E4/Trem2^ko^ mice (**d**). Shown are genes at *p* < 0.05 cutoff. Heatmaps and line patterning graphs of DAM and homeostatic microglia genes that are upregulated in either APOE4 (**e-f**) or in APOE3 mice (**g-h**) mice in a *Trem2* and APOE-isoform dependent manner. Genes of interest are marked with colored circles and shown as line patterning

The lightcyan module was highly and negatively correlated to *Trem2* status in all genotypes regardless of amyloid deposition or APOE isoform, indicating a decreased expression of these genes in all Trem2^ko^ mice (Fig. 4b). This module represented processes such as immune response, innate immune response, inflammatory response, integrin-mediated signaling pathway, phagocytosis, and chemotaxis (Fig. 4c, Suppl. Table 1). The top hub genes (i.e. the most interconnected genes) in lightcyan module were *Csf1r*, *Mpeg1*, *Cx3cr1*, *Hexb,* and *Vsir* and were used to generate a representative network (Fig. 4d-e). This module is highly enriched in microglial-specific genes (48 out of 99 genes are microglia specific genes), indicating a strong impact of *Trem2* deficiency on microglial gene expression. As shown on the heat maps in Fig. 4f-h, the gene list of the lightcyan module is comprised of 26 DAM genes (such as *Clec7a*, *Cst7*, *Cd68*, *Itgax*/CD11c, *Mpeg1*), 36 homeostatic genes (*P2ry12*, *P2ry13*, *Cx3cr1*, *Itgam*/CD11b, *Tgfb1*), and a group of 37 genes (*Spi1*/PU.1, *Runx1*, *Treml2*, *Vsir*) not associated with DAM or homeostatic microglia. These genes are downregulated in Trem2^ko^ mice in all of the four respective genotypes and represent the common signature of *Trem2* deficiency. Interestingly, two important DAM genes, *Apoe* [17, 19] and *Tyrobp* [17], were not present in this module. The reason *Apoe* was missing from the Trem2 signature list of genes is that as shown on Fig. 1l, it was upregulated only in APP/E4 mice vs APP/E3 in response to the higher level of amyloid pathology in these mice (also see Suppl. Fig. 5B, Suppl. Table 3). *Tyrobp* was uniquely downregulated only in APP/E3/Trem2^ko^ vs APPE3 mice as well as in E3/Trem2^ko^ vs E3 mice (Suppl. Table 2). Furthermore, *Tyrobp* (a member of turquoise module) had a higher expression level in APP/E3 and E3 mice vs their APOE4 counterparts (see Fig. 5g, Suppl. Table 3).

To associate the Trem2 signature to the phenotype of the APP mice, we correlated the gene expression levels of the lightcyan module to amyloid deposition. As shown in Fig. 4i-j, percent coverage of 6E10 and X34 in the mice expressing wild-type *Trem2* correlated significantly and positively to the lightcyan module indicating that this module, enriched in microglial-specific genes, represents the transcriptional response to increasing amyloid deposition. In contrast, there was no significant correlation in APP/E3/Trem2^ko^ or APP/E4/Trem2^ko^ mice between amyloid deposition and lightcyan module eigengene expression, demonstrating that as deposition increases in Trem2 deficient APP mice, there is no corresponding increase of microglial gene expression suggesting that *Trem2* deletion blocks the normal response of microglia to the increased pathology.

### APOE isoform-specific effect on gene expression

Since we found a significant APOE isoform-specific effect (Fig. 4a, Suppl. Fig. 4, and Suppl. Table 1), we determined differentially expressed genes that are characteristic for each genotype by comparing brain transcriptome of APP/E4 vs APP/E3 mice, E4 vs E3 mice as well as their respective Trem2^ko^ counterparts (Fig. 5a-d, Suppl. Table 3). We observed more than twice as many differentially expressed genes when comparing APP/E4 vs APP/E3 mice (Fig. 5a) than in APP/E4/Trem2^ko^ vs APP/E3/Trem2^ko^ mice (Fig. 5b). Finding a high number of differentially expressed genes in brain transcriptomes of APP/E4 vs APP/E3 mice is expected because it reflects the difference in amyloid pathology that elicits a stronger response in APP/E4 than in APP/E3 mice. However, a reduced number of differentially expressed genes in APP/E4/Trem2^ko^ vs APP/E3/Trem2^ko^ mice, at approximately the same level of neurodegeneration as in the wild-type *Trem2* mice, suggests that *Trem2* deficiency impairs the normal response to the disease progression and “blunts” the differences between the transcriptomes.

We next searched for DAM and homeostatic genes that are significantly affected in Trem2 and APOE isoform-dependent manner in APP mice. Figure 5e-f shows heatmaps of DAM and homeostatic genes that have a higher expression level in APP/E4 vs APP/E3 mice and are affected by *Trem2* deficiency. As shown on the line patterning graphs (Fig. 5e-f) some of these, such as DAM genes *Clec7a*, *Itgax,* and *Mpeg1* or homeostatic *Pik3cg* gene, are part of the Trem2 signature and are downregulated in all Trem2^ko^ mice. However, they still retain a higher level of expression in APP/E4/Trem2^ko^ vs APP/E3/Trem2^ko^ as in APP/E4 vs APP/E3 mice, suggesting that these genes respond, at least to a degree, to the more advanced level of neurodegeneration in APP/E4 mice even as *Trem2* is absent. Another group of DAM genes (*Slc15a3*) and homeostatic genes (*Csf1r, Itgam/*Cd11b*, Laptm5,* and *Nckap1l*) had a significantly higher expression in APP/E4 vs APP/E3 mice but this difference disappeared between their Trem2^ko^ counterparts, suggesting an increased dependence on the presence of *Trem2*. Similarly, we identified *Trem2*-dependent genes with significantly higher expression in APP/E3 vs APP/E4 mice that failed to elicit the same response when *Trem2* was deleted. A few examples are shown on Fig. 5g-h: DAM associated (*Tyrobp, Cd37, Cyba,* and *Ctss*) and homeostatic genes (*C1qa, C1qb,* and *C1qc*).

In addition, we also identified APOE isoform-specific genes that were not associated with *Trem2* deficiency or amyloid pathology (Suppl. Fig. 5A, Suppl. Table 3). Among the genes upregulated in APOE4 mice were several members of *Serpina3* family (*Serpina3h*, *Serpina3k*, *Serpina3m*, *Serpina3n*), as well as *Ptprh*, *Abcg1*, and *Picalm* which have all previously been reported by us [39] and others [40]. All of *Serpina3* genes and *Ptprh* were upregulated and statistically significant in every E4 vs E3 comparison (APP/E3 vs APP/E4, APP/E3/Trem2^ko^ vs APP/E4/Trem2^ko^, E3 vs E4, and E3/Trem2^ko^ vs E4/Trem2^ko^) confirming that they were neither TREM2 nor amyloid dependent but strictly APOE isoform dependent.

### FISH identifies alterations in microglial gene expression within the plaque microenvironment as a result of *Trem2* deficiency and APOE isoform

**Fig. 6:**
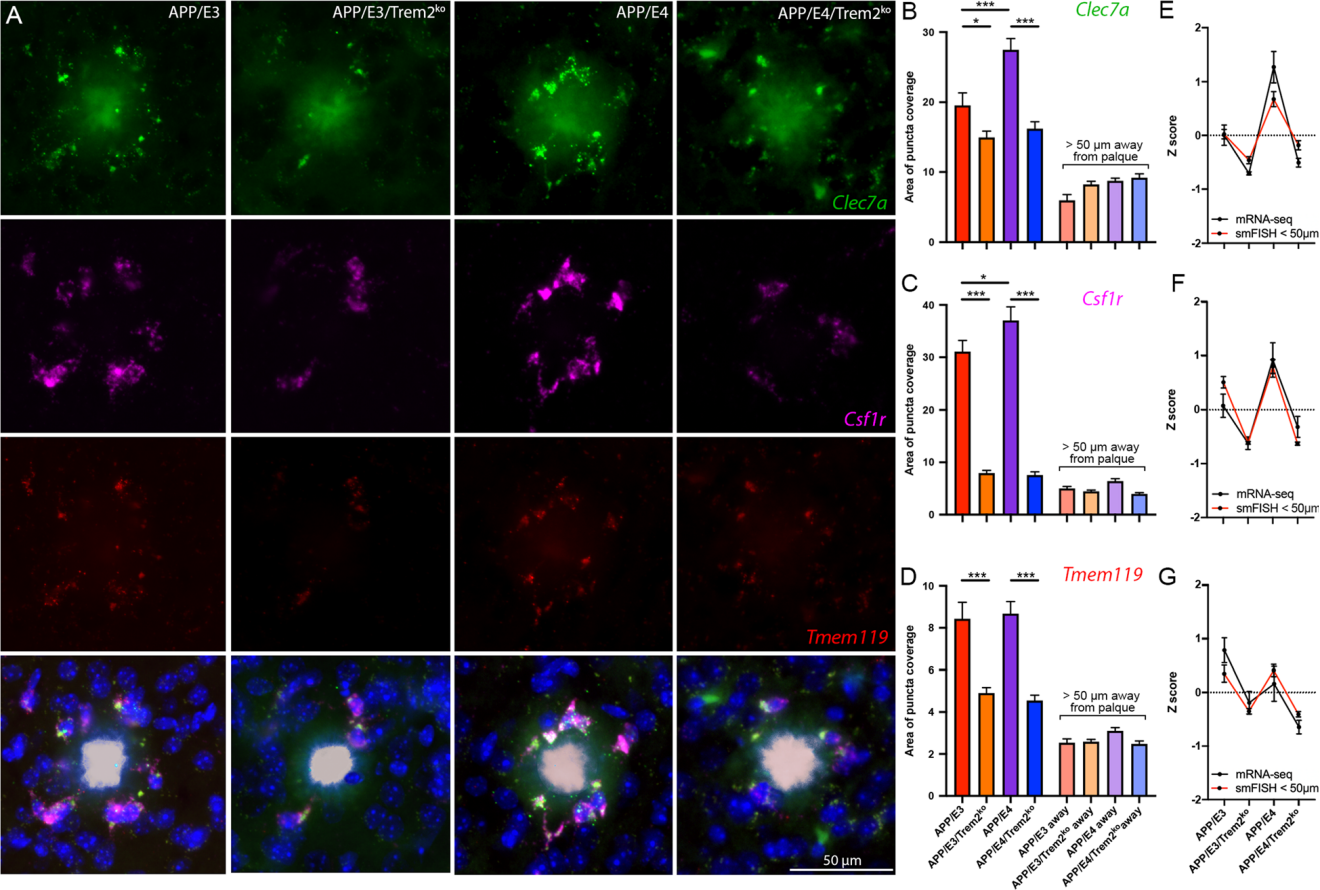
FISH identifies alterations in gene expression within plaque microenvironment as a result of *Trem2* deficiency. **a** Representative images of FISH analyses of microglia gene expression near amyloid plaques at 6.5 months of age (*Tmem119* – red, *Csf1r* – Pink, *Clec7a* – Green, Nuclei – Blue, Amyloid plaque - White). The area occupied by the puncta was quantified adjacent to plaques (< 50 μm) as well as away from plaques (> 50 μm) for APP/E3, APP/E3/Trem2^ko^, APP/E4, and APP/E4/Trem2^ko^ mice. Bar plots showing the area of puncta coverage adjacent to and away from plaques, and line patterning depicting the Z-score of RNA-seq data and FISH data together for *Clec7a* DAM marker (**b** & **e**), *Csf1r* homeostatic microglia marker (**c** & **f**), and *Tmem119* homeostatic microglia marker (**d** & **g**). FISH analysis by one-way ANOVA followed by Tukey’s multiple comparison test. *n* = 4 mice per group (equal males and females), an average of 86 plaques analyzed per genotype, bars represent mean ± SEM, and *** *p* < 0.001; * *p* < 0.05 using Tukey’s multiple comparison testing.

Our next goal was to validate RNA-seq data and characterized the spatial distribution of expressed mRNAs of three genes in relation to compact amyloid plaques at 6.5 months of age. We chose three genes that are part of Trem2 signature– two significantly affected homeostatic genes (*Tmem119* and *Csf1r*), and one significantly affected DAM gene (*Clec7a*) (Fig. 6). We selected these genes because *Tmem119* is a microglia-specific gene and *Csf1r* was the most connected hub gene in the network (Fig. 4e) and *Clec7a* was the top down-regulated gene in all Trem2 deficient mice. We performed FISH using RNAscope probes, coupled with histological detection of X34 positive Aβ plaques. We found that in the microenvironment surrounding plaques (< 50 μm), mRNA expression of all three genes was significantly decreased in both APP/E3/Trem2^ko^ and APP/E4/Trem2^ko^ mice when compared to their wild-type *Trem2* counterparts (Fig. 6b-d). In confirmation to RNA-seq results, *Csf1r* and *Clec7a* expression was also significantly higher in APP/E4 vs APP/E3 mice and the expression of *Tmem119* was not affected in APOE isoform-specific manner. This suggests that in addition to being *Trem2* dependent, *Csf1r* and *Clec7a* are also affected by APOE isoform as shown above on Fig. 5e-f. In contrast, there was no difference in gene expression of any of the analyzed genes away from the plaques (> than 50 μm away). The most probable explanation for the spatial difference in gene expression between Trem2^ko^ and wild-type Trem2 mice is the significant reduction in microglia recruitment around plaques in Trem2 deficient mice (Fig. 1f). We then compared the magnitude of the effect seen in the RNA-seq and FISH data using Z-scores to normalize each dataset to comparable levels. The FISH data collected within 50 μm of plaque center parallels the expression profile seen by RNA-seq (Fig. 6e-g). Thus, this experiment validated RNA-seq result confirming TREM2 and APOE isoform-specific effects on gene expression.

## Discussion

In the present study, we investigated the effect of *Trem2* deletion on the phenotype of APP transgenic mice expressing human APOE3 or APOE4 isoforms. We show that the absence of *Trem2* exacerbated cognitive impairments in APP transgenic mice but not in their non-APP littermates. Examination of the behavioral data showed that APP/E4 mice performed at the lower limit in these tasks and we were unable to observe a significant reduction in their Trem2^ko^ counterparts. The behavioral data demonstrate that APOE isoform impacts memory significantly more than Trem2 status, which is not surprising considering the higher impact of APOE isoform compared to TREM2 variants on the risk of late-onset AD [1, 2, 5–8]. These data are consistent with previous studies showing that increased human *TREM2* gene dosage in 5XFAD mice improved contextual fear conditioning memory [41]. In contrast, another study using the same AD model (5XFAD mice) has shown no impact of *Trem2* deficiency on spatial learning [26]. The observed diminished cognitive performance could be associated with the lack of microglial barrier around plaques and increased neuronal dystrophy observed in the TREM2 deficient mice. This is in agreement with previous data that showed *Trem2* haplodeficiency diminished the plaque-associated microglial barrier resulting in severe neuronal dystrophy [42].

The examination of amyloid plaque load revealed that while APOE isoform was a significant factor, *Trem2* deletion resulted in no change in steady-state plaque level in either APOE genotype at 6.5 months of age. This is an age where APP/E3 mice are characteristic of early Aβ pathology and APP/E4 mice exhibit a more advanced stage. Many of the studies aimed at better understanding the link between TREM2 function and AD have focused on amyloid plaque pathology however, with conflicting results. Using different AD models (5xFAD), *Trem2* deletion was shown to increase Aβ pathology during the very advanced stages of plaque pathology in a region-specific manner [9, 26]. Other studies demonstrated that *Trem2* deletion reduced plaque load early but increased it later with the disease progression [22, 41]. Conversely, amyloid PET imaging of the same AD model (APPPS-21) revealed that *Trem2* knockout resulted in accelerated fibrillar amyloid early, which equalized during the later stages of pathogenesis [24]. Our study is unique in that we utilized AD model mice expressing human APOE isoforms. We identified significant differences in the ratio of compact plaques to the protofibular halo amyloid staining, indicating a *Trem2* mediated effect on plaque compaction in agreement with previous reports [9, 23].

There are several aspects of TREM2 function that could explain a decreased ability of Trem2^ko^ microglia to perform their function. The loss of TREM2 cell-surface signaling may lead to a diminished capacity of microglia to recognize Aβ followed by a decreased uptake. Furthermore, the reduced numbers of microglia around amyloid plaques suggest that microglial chemotaxis was inhibited in APP expressing / Trem2^ko^ mice, thus restricting microglia movement towards Aβ, or any other damage in surrounding areas. As a consequence, the rate of plaque growth strongly correlated to the significant reduction of microglia barrier around the plaques (Figs. 2 and 3). Our transcriptomic data support this hypothesis, demonstrating a downregulation of genes associated with both phagocytosis and chemotaxis in Trem2^ko^ versus wild-type Trem2 mice (Fig. 4).

The novelty of our study is that we tested the effect of APOE isoform as an additional factor that could interact with *Trem2* to contribute to neurodegeneration. Previously, it was shown that, following microglial depletion, microglia derived APOE protein is reduced in Trem2^ko^ mice [24]. Here we have examined *Trem2* effect in an APOE isoform-dependent and amyloid-dependent manner by directly comparing age-matched APP/E3 and APP/E4 mice that are at different stages of amyloid pathology. We observed a difference in the number of microglia but not astrocytes (Fig. 1e-g), as well as APOE protein surrounding plaques between APP/E3 and APP/E4 mice that was not recapitulated in their Trem2^ko^ counterparts (Fig. 1j-k). Similar pattern of *Apoe* mRNA expression was detected by RNA-seq in APP and their WT littermates, confirming that *Apoe,* as a DAM gene [17], responds to the differences in the amyloid pathology in APP/E4 vs APP/E3 mice (Fig. 1l and Suppl. Fig. 5B). Furthermore, our results suggest the effect of *Trem2* deletion on *Apoe* expression is apparent only in APP mice and depends on the amyloid deposition. APOE is secreted mainly by astrocytes and less by microglia, but since *Trem2* is microglia-specific gene, it is reasonable to expect that *Trem2* deletion will directly affect *Apoe* expression in microglia. In order to identify microglia, we used the microglia-specific gene *Tmem119* [43, 44]. It should be noted that *Tmem119* is a homeostatic gene and in our study its expression was decreased by *Trem2* deficiency. However, as in previous studies [43, 44] we used it only as a marker to label microglia without assessing its expression. Our results using in situ hybridization confirmed that microglial *Apoe* depends on *Trem2* presence and is decreased in APP/E4/Trem2^ko^ microglia vs APP/E4 counterparts (Fig. 1m-n).

Our data also indicate that the effect of *Trem2* deficiency on plaque growth is stronger at earlier stages of amyloid deposition when plaques are smaller. Considering that APP/E4 mice show earlier onset of amyloid pathology, the TREM2 effect on plaque growth in mice expressing this isoform is observed in mice younger than their APOE3 counterparts. A possible explanation for the observed APOE isoform effects is the difference in their abilities to transport lipids and cholesterol [45] that can impact APOE receptor-binding properties. Recently, we reported a significant difference in brain phospholipid content of APOE2, E3, and E4 in AD patients [46] and APOE4-containing lipoproteins were shown to be less lipidated than APOE3 [47]. Thus, APOE4 may impede Aβ phagocytosis via reduced affinity for receptor binding (including TREM2 receptor), or changes in proteolytic degradation of Aβ (reviewed in [3, 48]).

There is an apparent inconsistency between the role of TREM2 in plaque growth, albeit at different ages for APP/E3 and APP/E4, and its lack of effect on steady-state amyloid load in APP/Trem2^ko^ mice. This could be explained by the balance between Aβ clearance mechanisms at different phases of amyloid pathology, i.e. microglia phagocytosis vs efflux via the BBB. At the earlier stages of amyloid deposition, the prevailing Aβ species in brain interstitial fluid are monomers and low molecular weight oligomers that are easily cleared out of the brain by efflux via BBB with half-life 1.5 h [30, 49]. In contrast, with the progression of the pathology, there is an increase of high molecular weight Aβ oligomers [50] leading to the increase of Aβ half-life in interstitial fluid [49, 51]. Thus, in mice with significant amyloid pathology, the faster BBB clearance mechanism is impeded and defects in microglia-mediated clearance mechanisms result in an increase of its deposition into plaques (Suppl. Fig. 6).

The lightcyan module represents the Trem2 signature which is enriched for microglial-specific genes indicating that *Trem2* deficiency has a robust effect on microglial gene expression. Because of the limitations of bulk RNA-seq data, this conclusion needs to be confirmed by single cell RNA-seq data. The main hubs of lightcyan module network are *Csf1r*, *Mpeg1*, *Cx3cr1*, *Hexb* and *Visr*. Previously, few of these genes such as *Cx3cr1*, *Hexb*, *P2ry12*, *P2ry13*, and *Siglech* were classified as *Trem2* dependent [17]. However, as part of the Trem2 signature, we also identified a group of genes which had never before been linked to *Trem2* deficiency, including *Spi1*/PU.1 [52], *Adgre1* [53]*, Ctsc* [54], *Fcgr1* [55], *Cd68* [56]*,* and the hub gene *Visr* [57]*.* These genes are enriched in immune cells [58] and follow similar expression patterns in microglia expressing either APOE isoform. Our topmost downregulated genes in Trem2^ko^ mice in all four sets of comparisons were *Clec7a* and *Itgax*. Both, *Clec7a* and *Itgax* have been previously identified as drivers of a “primed” microglia phenotype associated with neurodegeneration and aging [59]. We found no change in a few genes, previously found downregulated in Trem2^ko^ mice, namely *Axl, Csf1,* and *Spp1* [19, 26, 41, 60]. It should be noted that the Trem2 signature as identified in this study, incorporates only the genes that are commonly affected by *Trem2* deficiency in APP as well in their WT littermates. DAM genes that respond mainly to the increased neurodegeneration such as *Apoe* and *Lpl* were not identified as part of the common Trem2 signature. Both *Apoe* and *Lpl* were upregulated only in APP/E4 mice vs APP/E3 in response to the higher level of amyloid pathology seen in these mice (Suppl. Table 3).

Interestingly, *Tyrobp* implicated in the TREM2 checkpoint [17] was downregulated in Trem2^ko^ mice in an APOE isoform-specific manner in both APP/E3 mice and in their WT non-APP littermates (see Fig. 5g). The most probable reason is that we are exploring an effect of *Trem2* deletion in mice expressing human *APOE* instead of mouse *Apoe* and as mentioned above the differences in APOE3 and APOE4 lipidation could affect receptor binding and signal transduction pathways reflecting on brain transcriptome. In addition to *Tyrobp*, we identified as uniquely upregulated in APP/E3 vs APP/E3/Trem2^ko^ mice, i.e. in an APOE3-*Trem2* dependent manner, several genes involved in the C1q complement cascade. *Tyrobp* has been previously regarded as a regulator of genes involved in the complement pathway [61, 62], and is part of a predicted protein-protein interaction network along with *C1qa*, *C1qb*, *C1qc,* and *Ctss* [63].

We have previously shown an APOE4 isoform-specific increase of several *Serpina3* genes and *Ptprh* in human APOE targeted replacement mice [39]. In the current study, we confirmed that four members of *Serpina3* family (*Serpina3h*, *Serpina3k*, *Serpina3m*, *Serpina3n*), as well as *Ptprh,* are increased in both APP/E4 vs APP/E3 mice and their non-transgenic littermates (E4 vs E3) suggesting that their expression was not affected by amyloid deposition. We also established that the expression of the *Serpina3* genes was not affected by *Trem2* deficiency (see Suppl. Table 3). Recently, Zhao et al. [40] demonstrated a transcriptional upregulation of several genes from *Serpina3* family in the same APOE4 vs APOE3 mice. They also reported that the expression level of *SERPINA3* (human ortholog of *Serpina3n*) is higher in APOE4 carriers vs non-carriers, but is not significantly different when adjusted by AD status [40]. Interestingly, in a recent study examining the effect of APOE isoform on the transcriptome in human AD cortex (right inferior parietal lobule), we found that the expression of *SERPINA3*, as well as *PTPRH,* was significantly higher in APOE2 carriers vs APOE4 carriers [46]. The APOE isoform-dependent effect on the expression of members of this gene family in human and mouse warrants further research.

## Conclusion

In conclusion, the results of this study provide insight into the complex effect of TREM2 on phenotype, and brain transcriptomes in mice expressing human APOE isoforms. We show that the absence of *Trem2* exacerbated cognitive impairments in APP transgenic mice but not in their WT littermates. *Trem2* deletion significantly reduced microglia barrier around the plaques in correlation with the increased plaque growth rate. The differences in expression levels identified a Trem2 signature - a cluster of highly connected immune response genes, commonly downregulated as a result of *Trem2* deletion and regardless of the APOE isoform. Surprisingly, the lack of TREM2 significantly decreased *Apoe* mRNA expression in APP/E4 but not in APP/E3 mice a result that was confirmed by APOE protein analysis. Future studies are needed to better understand the role of TREM2 through the normal aging and in microglial response to neuronal injury and amyloid deposition.

## Supplementary information

**Additional file 1: Supplemental Figure 1** (supplemental to Fig. 1). No significant differences in locomotor activity, learning during novel object recognition and fear conditioning, and Aβ ELISA, as a result of *Trem2* deletion. Cued fear conditioning in APP mice (A & B) and all behavioral analysis for wild-type controls showed no significant effect of APOE or *Trem2*. There was no significant difference in percent freezing during the novel phase (C & D) or learning phase (E & F) of the contextual-cued fear conditioning (CCFC) for all experimental groups assessed. There was also no significant difference in total distance (m) traveled during the Open Field phase of NOR in APP/E3, APP/E4, APP/E3/Trem2^ko^, and APP/E4/Trem2^ko^ mice (G) or wild-type controls (H). *n* = 6–14 mice per group. For APP mice *n* = 6–7 mice/genotype/sex (12–14 mice/genotype). For non-APP mice, *n* = 4–7 mice/genotype/sex (8–14 mice/genotype). Analysis of cortical soluble Aβ (I) and cortical insoluble Aβ (J) ELISA levels by two-way ANOVA did not show an interaction between main factors: APOE and *Trem2*. There was a main effect of APOE isoform but not *Trem2* status. Sidak multiple comparisons test showed statistical significance between APP/E3 and APP/E4 mice. *n* = 14–22 mice per group (equal males and females). On the graphs, colored symbols indicate female and black symbols male mice.

**Additional file 2: Supplemental Figure 2** (supplemental to Fig. 1). The absence of *Trem2* similarly impacts plaque diffusivity but has no effect on steady-state amyloid load. (A) Representative images of 6E10 anti-Aβ immunostaining showing both diffuse and compact plaques (4X magnification). (B) Representative images of ThioS staining showing compact plaques (4X magnification). (C) 6E10-positive plaques were analyzed by two-way ANOVA showing no interaction between *Trem2* and APOE as factors. There was a significant main effect of APOE isoform (*p* < 0.0001), but no effect of *Trem2* deficiency. Sidak multiple comparisons test shows no significant differences between APP/E3 and APP/E3/Trem2^ko^ or between APP/E4 and APP/E4/Trem2^ko^ mice. *n* = 22–30 mice per group (equal males and females). (D) ThioS staining confirmed 6E10 staining results with no significant main effect of *Trem2* status or interaction. (E) Representative images of X34 and OC staining showing both diffuse and compact plaques (4X magnification). (F-G) X34 and OC staining confirmed 6E10 and ThioS staining results with no significant main effect of *Trem2* status or interaction for either X34 or OC. Sidak multiple comparisons test showed a statistical significance between APP/E3 and APP/E4 mice (*p* < 0.05). *n* = 14–16 mice per group (equal males and females). Colored dots represent female mice. (H) A visual depiction of what is counted as core-bound OC, total OC, and non-core bound OC used to generate data in Fig. 1d. (I) Representative images of individual X34 and OC labeled amyloid deposits. (J) Analysis of the OC/X34 ratio. *n* = 896–1569 plaques from 8 mice per group (equal male and female). For all histological analyses, one-way ANOVA was used followed by Tukey’s multiple comparison test. Bars represent mean ± SEM. *** *p* < 0.001; ** *p* < 0.01; * *p* < 0.05; NS, not significant.

**Additional file 3: Supplemental Figure 3** (supplemental to Fig. 2. In vivo plaque labeling using X04. (A) Schematic timeline of in vivo plaque labeling using an injection of X04 30 days prior to tissue harvesting. (B) Analysis of the plaques imaged from mice injected at 5.5 months and sacrificed 48 h later shows no difference in the volume of X04 and TR (*n* = 110 plaques). (C) Plotting TR against X04 shows minimal deviation from the expected 1:1 ratio (R^2^ = 0.9579), a growth volume near 0, and FC near 1. (D) Scatterplot wherein red dots denote the plaque growth rate in the 48-h control plaques (right axis), and the TR volume on the X axis. Black dots represent the entire experimental dataset binned by plaque size, with 94% of the plaques falling within the grey shaded box of the min and max values analyzed in the 48-h control plaques. (E) Representative confocal imaging of an amyloid plaque 48 h following X04 injection with IBA1 in green, X04 in blue, TR in red and the X04-TR merge in pink. Quantification of the volume of X04, TR and growth rate (TR-X04) in 4.5-month-old mice (F) and 6.5-month-old mice (G). (H) Representative images depicting how analysis metrics were derived. Confocal images were loaded into Imaris and 3D renderings generated for X04 and TR to calculate the volume. Plaque growth rate was calculated by subtracting the volume of the plaque at the time of in vivo labeling (X04, blue) from the volume of the plaque at the time of sacrifice (TR, red). 3D renderings were created to assess IBA1 (green) colocalization with the surface of the TR plaque. The plaque surface area contacted by microglia (yellow) is subtracted from the total surface area (grey) to quantify the exposed surface area of each plaque (purple, the surface area not covered by microglia). Analysis by one-way ANOVA followed by Tukey’s multiple comparison test. Bars represent mean ± SEM. * *p* < 0.05; ** *p* < 0.01; *** *p* < 0.001.

**Additional file 4: Supplemental Figure 4** (supplemental to Fig. 4). WGCNA identifies patterns of gene expression characteristic to each of the eight experimental groups. (A) WGCNA was used to identify correlations between gene expression and each of the 8 genotypes: APP/E3, APP/E3/Trem2^ko^, APP/E4, APP/E4/Trem2^ko^ and their corresponding non-APP counterparts (E3, E3/Trem2^ko^, E4, E4/Trem2^ko^). Numbers on the heatmap represent Pearson correlation and *p*-value in parenthesis. Modules of interest are marked with red circles. (B) The dendrogram visualizes the relative similarity between identified modules, with modules that appear close to each other having a more similar expression profile. Heatmap of the Pearson correlation coefficient between each module. (C) Gene expression heatmap and bar plots for each animal from turquoise – correlates positively to all APOE3 mice, as well as a network generated from top 3 hub genes (D) and GO term bar plots indicate the -log10P value for each term. The associated point in the center of each bar represents the percent of submitted genes found in each GO term (E). (F-H) Heatmap, bar plots, network, and GO terms for the lightyellow module – correlates positively to all APOE4 mice.

**Additional file 5: Supplemental Figure 5** (supplemental to Fig. 5). The expression of *Serpina3* family is higher in APOE4 than in APOE3 mice and cell type specific differentially expressed genes. (A) Bar plots of *Serpina3h*, *Serpina3k*, *Serpina3m*, and *Serpina3n* from the same 6.5-month-old WT and APP mice as shown on Figs. 4 and 5. (B) Bar plots depicting the average *Apoe* gene expression in APOE3 and APOE4 mice as identified by RNA-seq and statistics generated using edgeR.

**Additional file 6: Supplemental Figure 6.** Suggested model, illustrating the impact of *Trem2* deletion on the phenotype and transcriptome in APP/E3 and APP/E4 mice. (A) Lack of *Trem2* does not impact steady state amyloid deposition, impacts plaque growth, reduces microglia reactivity and worsens behavior in APP/E3/Trem2^ko^ and APP/E4/Trem2^ko^ mice as compared to their *Trem2*-expressing counterparts. Arrows are relative to their Trem2-expressing counterparts. (B) Differential effects of *Trem2* deficiency on microglia transcriptome in the same mice. a) Topmost affected Trem2 signature genes; b-c) Examples of Trem2-APOE dependent genes with expression higher in APP/E4 mice (b) or APP/E3 mice (c). (C) A graphical hypothesis regarding the importance of microglia barrier on the accumulation of Aβ and plaque dynamics. (a) and (c), In the early stage of amyloid deposition low molecular weight Aβ species are prevailing in interstitial fluid and are cleared mainly via efflux through the blood-brain barrier. (b) and (d), In the later stages of amyloid deposition, high molecular weight Aβ oligomers accumulate in interstitial fluid that impedes Aβ efflux via blood-brain barrier and microglia phagocytosis becomes a major component of Aβ removal. We hypothesize that there is increased reliance on functional *Trem2* on Aβ clearance in the late stages of amyloid pathology.

**Additional file 7: Supplemental Table 1**. WGCNA.

**Additional file 8: Supplemental Table 2**. Trem2-GO terms.

**Additional file 9: Supplemental-Table 3.** APOE-Isoform-GO terms.

## Data Availability

The RNA-seq expression data has been deposited in the GEO database under the accession number: GSE144125.

## Abbreviations

Aβ: Amyloid β
BBB: Brain Blood Barrier
CCFC: Contextual and Cued Fear Conditioning
DAM: Disease-associated microglia
FISH: Fluorescent in situ hybridization
GWAS: Genome-wide association studies
LOAD: Late-onset Alzheimer’s disease
NOR: Novel object recognition
TR: Thiazine red
WGCNA: Weighted gene co-expression network analysis
X34: Methoxy-X34
X04: Methoxy-X04
