# Supplementary figures and images for: Trem2 deficiency differentially affects phenotype and transcriptome of human APOE3 and APOE4 mice
Source: Mol Neurodegener. 2020 Jul 23;15:41. doi: 10.1186/s13024-020-00394-4 (PMC7379780; doi:10.1186/s13024-020-00394-4)

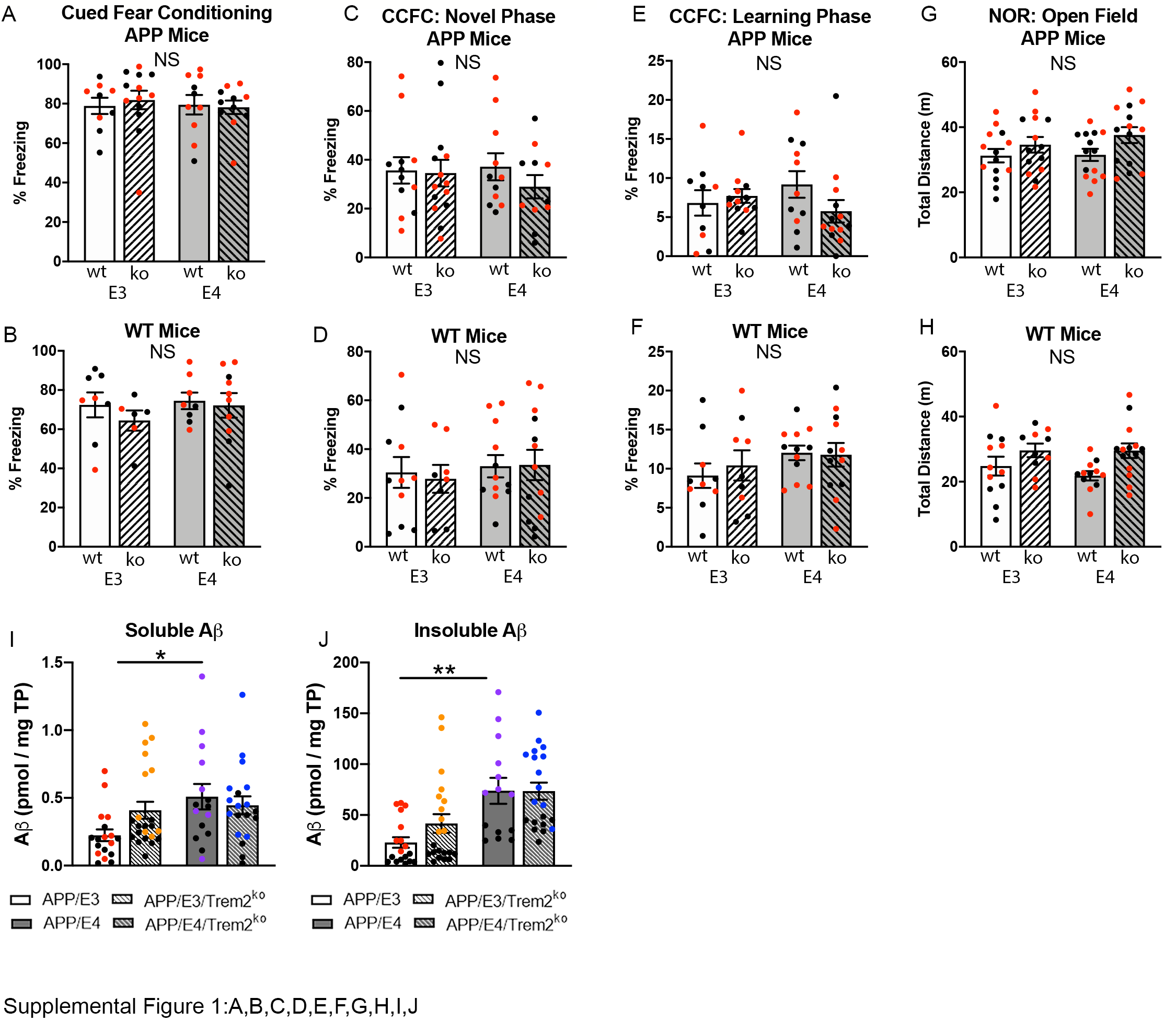

Supplement: Supplementary file 1 — Additional file 1: Supplemental Figure 1 (supplemental to Fig. 1). No significant differences in locomotor activity, learning during novel object recognition and fear conditioning, and Aβ ELISA, as a result of Trem2 deletion. Cued fear conditioning in APP mice (A & B) and all behavioral analysis for wild-type controls showed no significant effect of APOE or Trem2. There was no significant difference in percent freezing during the novel phase (C & D) or learning phase (E & F) of the contextual-cued fear conditioning (CCFC) for all experimental groups assessed. There was also no significant difference in total distance (m) traveled during the Open Field phase of NOR in APP/E3, APP/E4, APP/E3/Trem2ko, and APP/E4/Trem2ko mice (G) or wild-type controls (H). n = 6–14 mice per group. For APP mice n = 6–7 mice/genotype/sex (12–14 mice/genotype). For non-APP mice, n = 4–7 mice/genotype/sex (8–14 mice/genotype). Analysis of cortical soluble Aβ (I) and cortical insoluble Aβ (J) ELISA levels by two-way ANOVA did not show an interaction between main factors: APOE and Trem2. There was a main effect of APOE isoform but not Trem2 status. Sidak multiple comparisons test showed statistical significance between APP/E3 and APP/E4 mice. n = 14–22 mice per group (equal males and females). On the graphs, colored symbols indicate female and black symbols male mice. [file 13024_2020_394_MOESM1_ESM.tif]

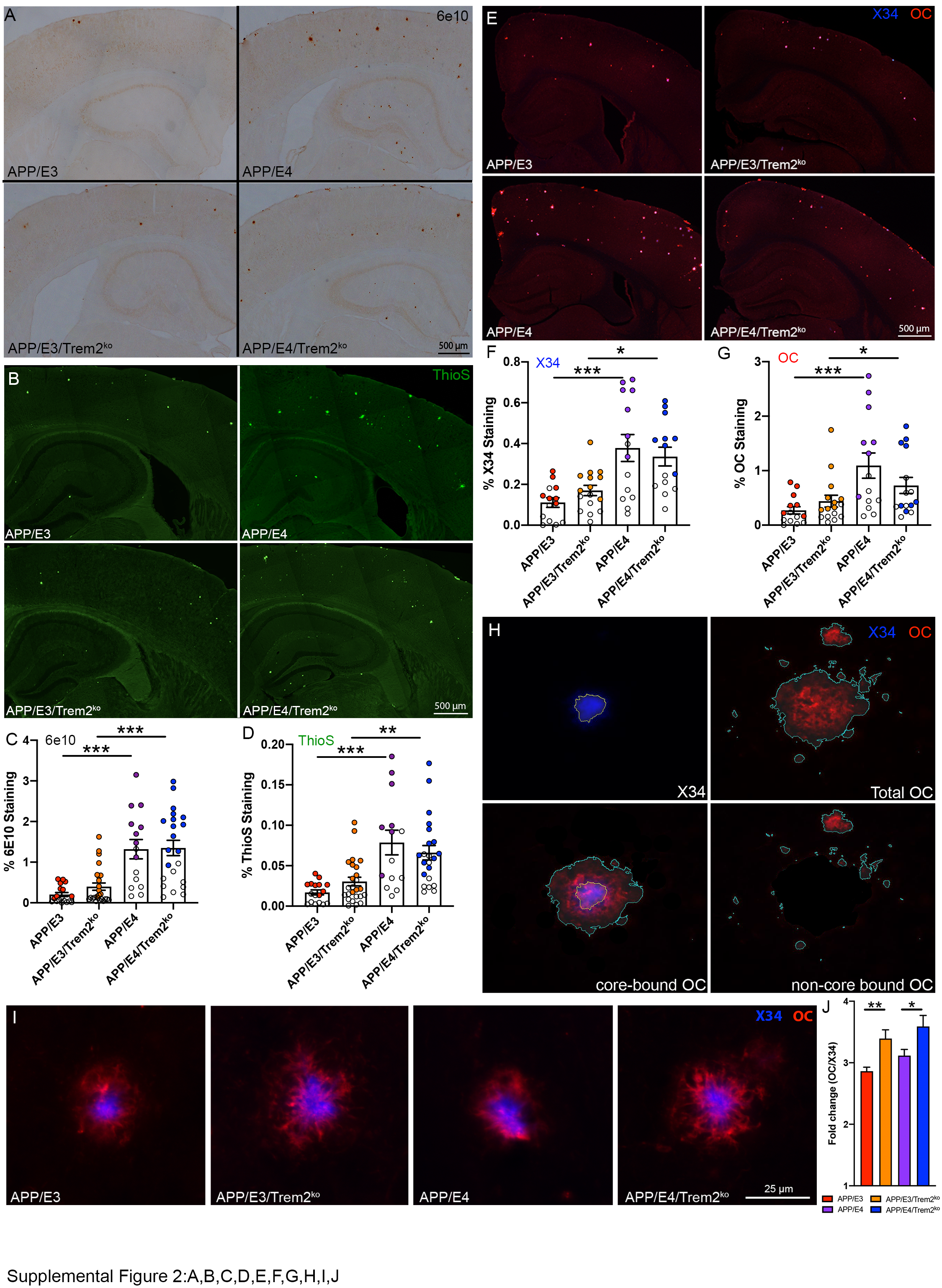

Supplement: Supplementary file 2 — Additional file 2: Supplemental Figure 2 (supplemental to Fig. 1). The absence of Trem2 similarly impacts plaque diffusivity but has no effect on steady-state amyloid load. (A) Representative images of 6E10 anti-Aβ immunostaining showing both diffuse and compact plaques (4X magnification). (B) Representative images of ThioS staining showing compact plaques (4X magnification). (C) 6E10-positive plaques were analyzed by two-way ANOVA showing no interaction between Trem2 and APOE as factors. There was a significant main effect of APOE isoform (p < 0.0001), but no effect of Trem2 deficiency. Sidak multiple comparisons test shows no significant differences between APP/E3 and APP/E3/Trem2ko or between APP/E4 and APP/E4/Trem2ko mice. n = 22–30 mice per group (equal males and females). (D) ThioS staining confirmed 6E10 staining results with no significant main effect of Trem2 status or interaction. (E) Representative images of X34 and OC staining showing both diffuse and compact plaques (4X magnification). (F-G) X34 and OC staining confirmed 6E10 and ThioS staining results with no significant main effect of Trem2 status or interaction for either X34 or OC. Sidak multiple comparisons test showed a statistical significance between APP/E3 and APP/E4 mice (p < 0.05). n = 14–16 mice per group (equal males and females). Colored dots represent female mice. (H) A visual depiction of what is counted as core-bound OC, total OC, and non-core bound OC used to generate data in Fig. 1d. (I) Representative images of individual X34 and OC labeled amyloid deposits. (J) Analysis of the OC/X34 ratio. n = 896–1569 plaques from 8 mice per group (equal male and female). For all histological analyses, one-way ANOVA was used followed by Tukey’s multiple comparison test. Bars represent mean ± SEM. *** p < 0.001; ** p < 0.01; * p < 0.05; NS, not significant. [file 13024_2020_394_MOESM2_ESM.tif]

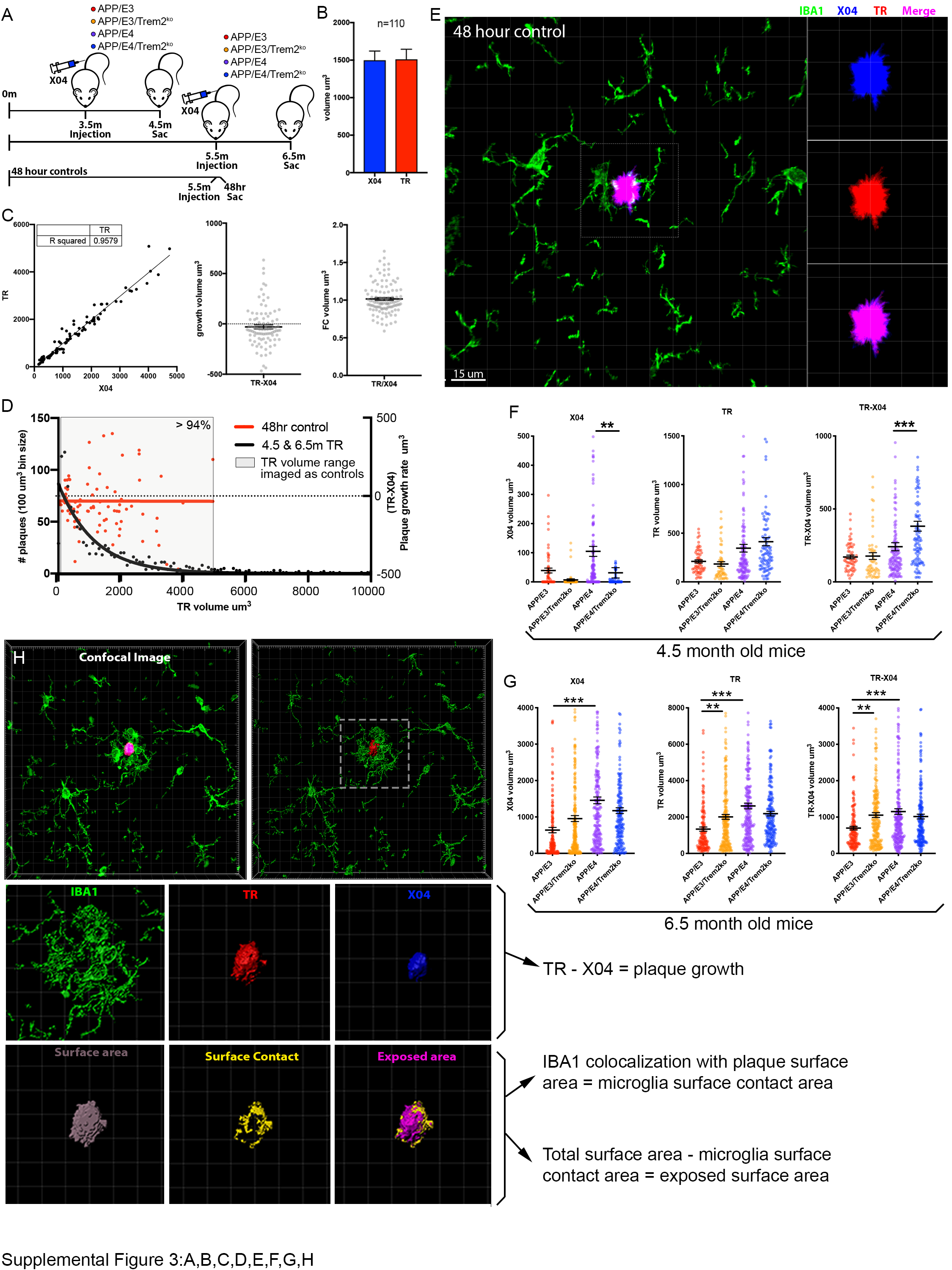

Supplement: Supplementary file 3 — Additional file 3: Supplemental Figure 3 (supplemental to Fig. 2. In vivo plaque labeling using X04. (A) Schematic timeline of in vivo plaque labeling using an injection of X04 30 days prior to tissue harvesting. (B) Analysis of the plaques imaged from mice injected at 5.5 months and sacrificed 48 h later shows no difference in the volume of X04 and TR (n = 110 plaques). (C) Plotting TR against X04 shows minimal deviation from the expected 1:1 ratio (R2 = 0.9579), a growth volume near 0, and FC near 1. (D) Scatterplot wherein red dots denote the plaque growth rate in the 48-h control plaques (right axis), and the TR volume on the X axis. Black dots represent the entire experimental dataset binned by plaque size, with 94% of the plaques falling within the grey shaded box of the min and max values analyzed in the 48-h control plaques. (E) Representative confocal imaging of an amyloid plaque 48 h following X04 injection with IBA1 in green, X04 in blue, TR in red and the X04-TR merge in pink. Quantification of the volume of X04, TR and growth rate (TR-X04) in 4.5-month-old mice (F) and 6.5-month-old mice (G). (H) Representative images depicting how analysis metrics were derived. Confocal images were loaded into Imaris and 3D renderings generated for X04 and TR to calculate the volume. Plaque growth rate was calculated by subtracting the volume of the plaque at the time of in vivo labeling (X04, blue) from the volume of the plaque at the time of sacrifice (TR, red). 3D renderings were created to assess IBA1 (green) colocalization with the surface of the TR plaque. The plaque surface area contacted by microglia (yellow) is subtracted from the total surface area (grey) to quantify the exposed surface area of each plaque (purple, the surface area not covered by microglia). Analysis by one-way ANOVA followed by Tukey’s multiple comparison test. Bars represent mean ± SEM. * p < 0.05; ** p < 0.01; *** p < 0.001. [file 13024_2020_394_MOESM3_ESM.tif]

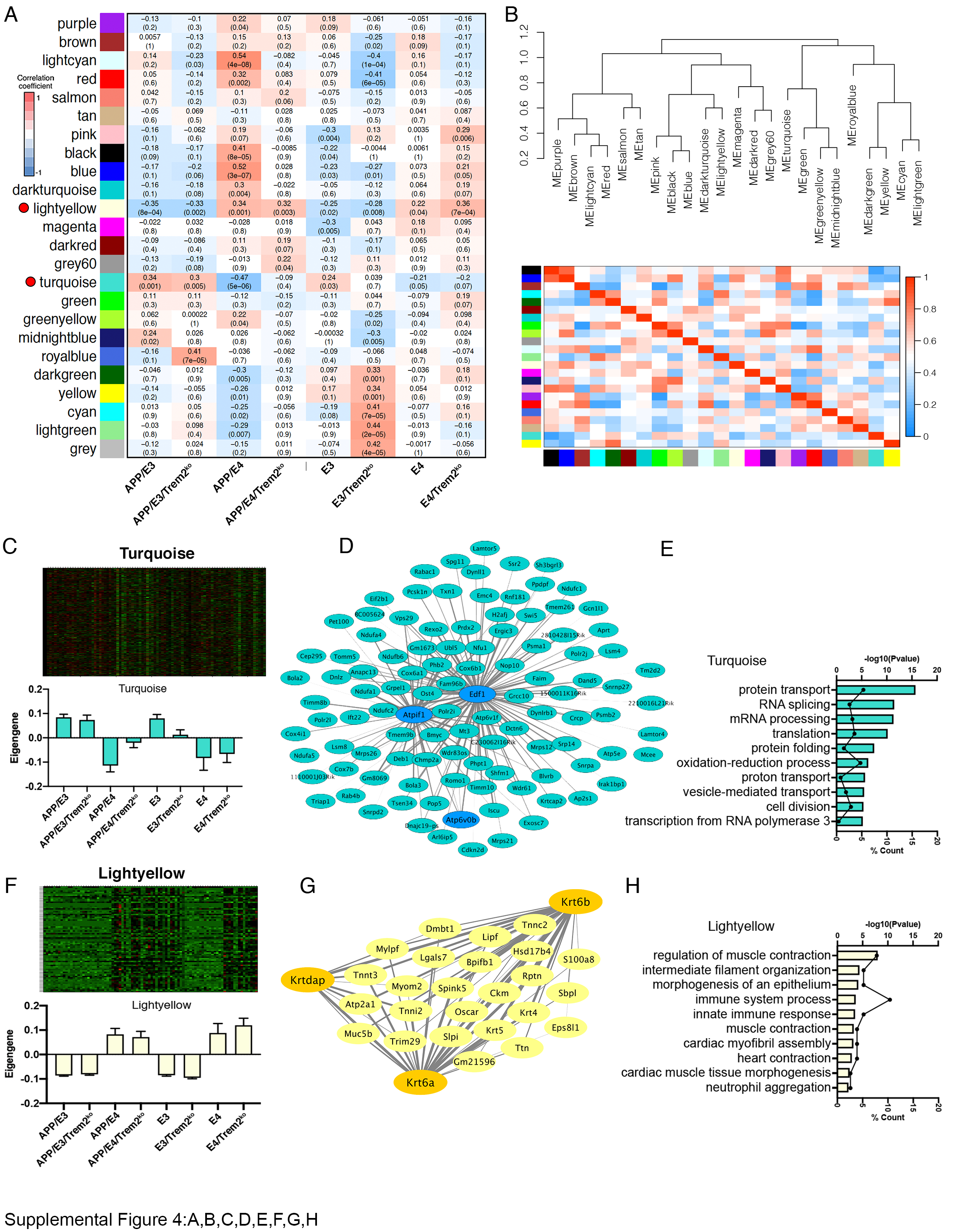

Supplement: Supplementary file 4 — Additional file 4: Supplemental Figure 4 (supplemental to Fig. 4). WGCNA identifies patterns of gene expression characteristic to each of the eight experimental groups. (A) WGCNA was used to identify correlations between gene expression and each of the 8 genotypes: APP/E3, APP/E3/Trem2ko, APP/E4, APP/E4/Trem2ko and their corresponding non-APP counterparts (E3, E3/Trem2ko, E4, E4/Trem2ko). Numbers on the heatmap represent Pearson correlation and p-value in parenthesis. Modules of interest are marked with red circles. (B) The dendrogram visualizes the relative similarity between identified modules, with modules that appear close to each other having a more similar expression profile. Heatmap of the Pearson correlation coefficient between each module. (C) Gene expression heatmap and bar plots for each animal from turquoise – correlates positively to all APOE3 mice, as well as a network generated from top 3 hub genes (D) and GO term bar plots indicate the -log10P value for each term. The associated point in the center of each bar represents the percent of submitted genes found in each GO term (E). (F-H) Heatmap, bar plots, network, and GO terms for the lightyellow module – correlates positively to all APOE4 mice. [file 13024_2020_394_MOESM4_ESM.tif]

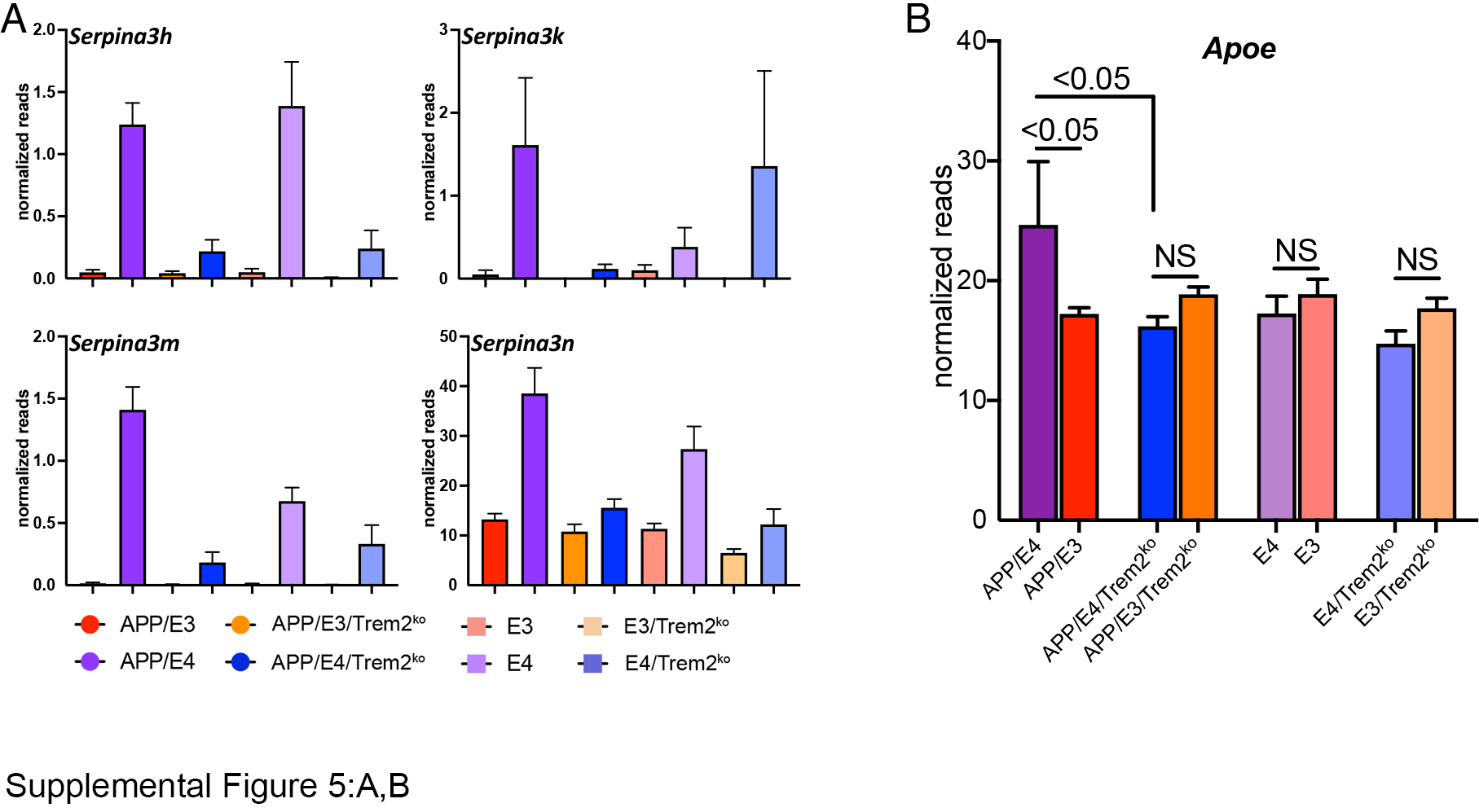

Supplement: Supplementary file 5 — Additional file 5: Supplemental Figure 5 (supplemental to Fig. 5). The expression of Serpina3 family is higher in APOE4 than in APOE3 mice and cell type specific differentially expressed genes. (A) Bar plots of Serpina3h, Serpina3k, Serpina3m, and Serpina3n from the same 6.5-month-old WT and APP mice as shown on Figs. 4 and 5. (B) Bar plots depicting the average Apoe gene expression in APOE3 and APOE4 mice as identified by RNA-seq and statistics generated using edgeR. [file 13024_2020_394_MOESM5_ESM.tif]

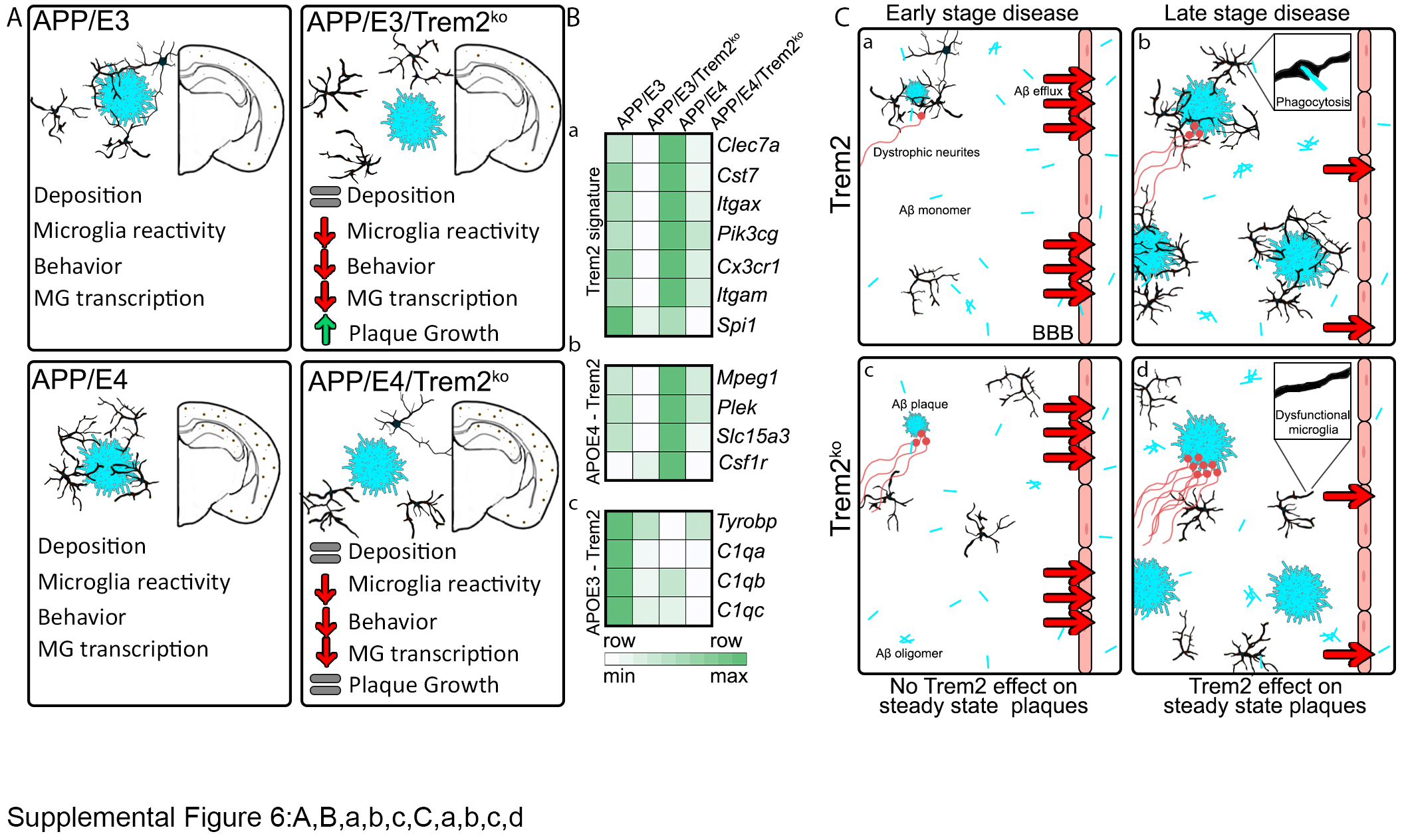

Supplement: Supplementary file 6 — Additional file 6: Supplemental Figure 6. Suggested model, illustrating the impact of Trem2 deletion on the phenotype and transcriptome in APP/E3 and APP/E4 mice. (A) Lack of Trem2 does not impact steady state amyloid deposition, impacts plaque growth, reduces microglia reactivity and worsens behavior in APP/E3/Trem2ko and APP/E4/Trem2ko mice as compared to their Trem2-expressing counterparts. Arrows are relative to their Trem2-expressing counterparts. (B) Differential effects of Trem2 deficiency on microglia transcriptome in the same mice. a) Topmost affected Trem2 signature genes; b-c) Examples of Trem2-APOE dependent genes with expression higher in APP/E4 mice (b) or APP/E3 mice (c). (C) A graphical hypothesis regarding the importance of microglia barrier on the accumulation of Aβ and plaque dynamics. (a) and (c), In the early stage of amyloid deposition low molecular weight Aβ species are prevailing in interstitial fluid and are cleared mainly via efflux through the blood-brain barrier. (b) and (d), In the later stages of amyloid deposition, high molecular weight Aβ oligomers accumulate in interstitial fluid that impedes Aβ efflux via blood-brain barrier and microglia phagocytosis becomes a major component of Aβ removal. We hypothesize that there is increased reliance on functional Trem2 on Aβ clearance in the late stages of amyloid pathology. [file 13024_2020_394_MOESM6_ESM.tif]
